# Differential expression and regulation of MS4A family members in myeloid cells in physiological and pathological conditions

**DOI:** 10.1002/JLB.2A0421-200R

**Published:** 2021-08-04

**Authors:** Rita Silva‐Gomes, Sarah N. Mapelli, Marie‐Astrid Boutet, Irene Mattiola, Marina Sironi, Fabio Grizzi, Federico Colombo, Domenico Supino, Silvia Carnevale, Fabio Pasqualini, Matteo Stravalaci, Rémi Porte, Andrea Gianatti, Constantino Pitzalis, Massimo Locati, Maria José Oliveira, Barbara Bottazzi, Alberto Mantovani

**Affiliations:** ^1^ Department of Biomedical Sciences Humanitas University, Pieve Emanuele Milan Italy; ^2^ IRCCS Humanitas Research Hospital, Rozzano Milan Italy; ^3^ ICBAS‐Institute of Biomedical Sciences Abel Salazar University of Porto Porto Portugal; ^4^ Instituto de Investigação e Inovação em Saúde and Instituto Nacional de Engenharia Biomédica Universidade do Porto Porto Portugal; ^5^ Centre for Experimental Medicine & Rheumatology, William Harvey Research Institute and Barts and The London School of Medicine and Dentistry Queen Mary University of London London UK; ^6^ Regenerative Medicine and Skeleton, RMeS, Inserm UMR 1229, Oniris, CHU Nantes Université de Nantes Nantes France; ^7^ Laboratory of Innate Immunity, Department of Microbiology, Infectious Diseases and Immunology Charité‐Universitätsmedizin Berlin, Campus Benjamin Franklin Berlin Germany; ^8^ Berlin Institute of Health (BIH) Berlin Germany; ^9^ Mucosal and Developmental Immunology Berlin Germany; ^10^ Infinity Université Toulouse, CNRS, Inserm, UPS Toulouse France; ^11^ Unit of Pathology Azienda Ospedaliera Socio Sanitaria Territoriale Papa Giovanni XXIII Bergamo Italy; ^12^ Department of Medical Biotechnologies and Translational Medicine University of Milan Milan Italy; ^13^ Department of Pathology and Oncology, Faculty of Medicine University of Porto Porto Portugal

**Keywords:** COVID‐19, monocytes/Mϕs, MS4A3, MS4A4A, MS4A6A, rheumatoid arthritis

## Abstract

The MS4A gene family encodes 18 tetraspanin‐like proteins, most of which with unknown function. MS4A1 (CD20), MS4A2 (FcεRIβ), MS4A3 (HTm4), and MS4A4A play important roles in immunity, whereas expression and function of other members of the family are unknown. The present investigation was designed to obtain an expression fingerprint of MS4A family members, using bioinformatics analysis of public databases, RT‐PCR, and protein analysis when possible. MS4A3, MS4A4A, MS4A4E, MS4A6A, MS4A7, and MS4A14 were expressed by myeloid cells. MS4A6A and MS4A14 were expressed in circulating monocytes and decreased during monocyte‐to‐Mϕ differentiation in parallel with an increase in MS4A4A expression. Analysis of gene expression regulation revealed a strong induction of MS4A4A, MS4A6A, MS4A7, and MS4A4E by glucocorticoid hormones. Consistently with in vitro findings, MS4A4A and MS4A7 were expressed in tissue Mϕs from COVID‐19 and rheumatoid arthritis patients. Interestingly, MS4A3, selectively expressed in myeloid precursors, was found to be a marker of immature circulating neutrophils, a cellular population associated to COVID‐19 severe disease. The results reported here show that members of the MS4A family are differentially expressed and regulated during myelomonocytic differentiation, and call for assessment of their functional role and value as therapeutic targets.

## Graphical Abstract

MS4A tetraspan molecules are differentially expressed and regulated in myeloid cells under normal and pathological conditions.

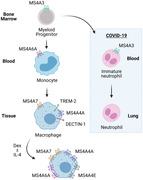

**Abbreviations**
AD: Alzheimer's disease
BALF: bronchoalveolar lavage fluid
cGMP: common granulocyte monocyte progenitor
ChIP‐Seq: chromatin immunoprecipitation sequencing
Chr: chromosome
DC: dendritic cell
DMARD: disease‐modifying antirheumatic drugs
FcεRIβ: Fc epsilon Receptor I
FLIM‐FRET: fluorescence lifetime imaging of Förster resonance energy transfer
GR: glucocorticoid receptor
GREs: glucocorticoid‐responsive elements
IHC: immunohistochemistry
KIT: receptor tyrosine kinase
MS4A: membrane‐spanning 4‐domains subfamily A
NK: natural killer
PKC: protein kinase C
RA: rheumatoid arthritis
RNA: ribonucleic acid
RNA‐seq: RNA sequencing
SARS‐CoV‐2: severe acute respiratory syndrome coronavirus‐2
scRNA‐seq: single cell RNA sequencing
SNPs: single nucleotide polymorphisms
SYK: spleen tyrosine kinase
TAM: tumor‐associated Mϕs
TPM: transcript per kilobase million.

## 1 INTRODUCTION

The *MS4A* gene family encodes for a highly hydrophobic group of proteins with 4 putative transmembrane domains (tetraspan) and several Protein Kinase C (PKC) phosphorylation sites.^1^, ^2^, ^3^ The first members of the family to be cloned were MS4A1 (CD20), MS4A2 (FcεRIβ), and MS4A3 (HTm4).^4^, ^5^, ^6^

**FIGURE 1:**
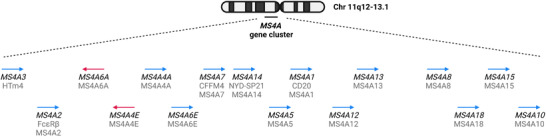
**Diagram of the MS4A genomic loci on chromosome 11**. Representation of the genomic location of each gene in chromosome 11. The names of the genes are depicted in black italic, and the protein names and colloquial names are depicted in grey. The blue and red arrows represent the genes located in different DNA strands (forward and reverse)

The human MS4A family currently comprises 18 genes, 16 of which are organized as a cluster on chromosome (Chr) 11q12^7^ (Fig 1). Two other genes, *TMEM176A* and *TMEM176B*, although having a different chromosomal location (Chr 7q36.1), share structural properties with MS4A members, and are considered part of the family.^7^ Members of this family are expressed by leukocytes (e.g., *MS4A1*, *MS4A2*, *MS4A3*, and *MS4A4A*), while others are present in non‐hematopoietic cells.^7^, ^8^, ^9^, ^10^ *MS4A1* (CD20) is a pan‐B cell marker^4^, ^11^ and monoclonal antibodies targeting this molecule treat B cell malignancies and autoimmunity.^12^, ^13^, ^14^, ^15^ *MS4A2* encodes the β‐chain of the high affinity IgE receptor (FcεRI) of basophils and mast cells, amplifying the intracellular signaling of this receptor,^16^, ^17^ and thus being involved in asthma and allergic disease.^18^ *MS4A3* was recently shown to mark early myeloid differentiation both in humans^19^ and mice,^20^ being highly expressed by common granulocyte‐monocyte progenitors (cGMP) in the human bone‐marrow.^19^ Indeed, this protein was shown to regulate the cell cycle, modulating the G1‐S phase transition.^21^, ^22^ MS4A4A is expressed by Mϕs^8^, ^9^ and further induced by M2‐like stimuli.^9^, ^23^, ^24^ Additionally, MS4A4A was also reported to be expressed by mast cells, playing an important role in the regulation of trafficking, signaling, and recycling of tyrosine kinase stem cell factor receptor (KIT),^25^ and in mediating FcƐRI signaling.^26^

In Mϕs, MS4A4A was shown to co‐localize with Dectin‐1 in lipid rafts and to be essential for the full activation of the Syk‐dependent signaling pathway of this innate immunity receptor upon ligand binding.^9^ Moreover, SNPs in the *MS4A4A* gene were shown to modulate the soluble TREM‐2 concentration in the cerebrospinal fluid of Alzheimer's disease (AD) patients, which increased levels are usually associated with reduced disease risk and delayed age at onset of disease.^27^ Indeed, genetic polymorphisms within the *MS4A* locus have been shown to be associated with different pathological conditions, including AD, gastric cancer, systemic sclerosis, progressive supranuclear palsy, or arthritis.^28^, ^29^, ^30^, ^31^, ^32^, ^33^ However, the contribution of these proteins to disease and the mechanisms underlying these associations are still unknown.

The results briefly summarized above indicate that, with the notable expression of CD20/MS4A1 and to some extent MS4A4A, knowledge of the expression and significance of MS4A members is scanty and fragmentary. These molecules belong therefore to the “ignorome”, that is the substantial part of the genome of unknown function.^34^, ^35^, ^36^ The present study was designed to obtain a fingerprint of the differential expression and regulation of MS4A family members in resting and activated human leukocyte populations under healthy and pathological conditions.

## 2 MATERIALS AND METHODS

### 2.1 Human monocyte‐derived Mϕs

Human monocytes were isolated from buffy coats or fresh blood from healthy donors by serial density‐gradient centrifugation as previously described,^23^ or using the CD14 MicroBead Kit (Miltenyi Biotec, Bergisch Gladbach, DE) accordingly to the manufacturer's instructions. Human blood cell isolation was approved by the institutional Ethical Committee of the Humanitas Clinical and Research Center (Authorization n° 2502, 09/04/2020 and Approval for the use of buffy coats issued on 28/01/2016). Monocytes were treated for 7 days with 25 ng/ml rhM‐CSF (Peprotech, London, GB) to obtain Mϕs, in RPMI 1640 (Lonza, Basel, CH) with 10% FBS (Lonza, Basel, CH) and 1% l‐glutamine (Lonza, Basel, CH). Mϕs were activated in vitro by 18 h incubation with 100 ng/ml 055:B5 LPS (Sigma–Aldrich, St Louis, MO, USA), 20 ng/ml rhIFN‐γ (Peprotech, London, GB), a combination of 100 ng/ml LPS and 20 ng/ml rhIFN‐γ, 20 ng/ml rhTNF (Peprotech, London, GB), 20 ng/ml rhIL‐10 (Peprotech, London, GB), 20 ng/ml rh TGF‐β (rhTGF‐β) (Peprotech, London, GB), 10^−6^ M Dexamethasone (Dex) (MP‐Biomedicals, Illkirch, FR), 20 ng/ml rhIL‐4, or 10^−6^ M Dex in combination with 20 ng/ml IL‐4.

### 2.2 Human blood leukocyte isolation

Freshly collected human blood was treated with 3% Dextran solution for red blood cells removal. PBMC and PMN were then isolated by Ficol‐density gradient centrifugation. PMN were further treated with ACK lysis buffer and prepared for fluorescence‐based cell sorting. In parallel, CD14 positive monocytes were purified from PBMC using CD14 MicroBeads (Miltenyi Biotec, Bergisch Gladbach, DE) accordingly to the manufacturer's instructions. The CD14 negative cell fraction was collected and prepared for fluorescence‐based cell sorting. Both PMN and CD14 negative cell fractions were incubated with Zombie Aqua Fixable Viability Kit (BioLegend, San Diego, CA, USA) for live/dead cell discrimination, followed by Fc receptor blocking with Human TruStain FcX™ (Biolegend, San Diego, CA, US). Finally, cells were incubated for 15 min at room temperature (RT) with directly conjugated antibodies. PMN were stained with anti‐CD16 (PerCP‐Cy5.5, Biolegend, San Diego, CA, USA) and anti‐CD66b (AF647, BD, Franklin Lakes, NJ, USA), while the CD14 negative PBMC fraction was stained with anti‐CD3 (BV605, BD Horizon, Franklin Lakes, NJ, US), anti‐CD19 (BV421, BD, Franklin Lakes, NJ, US), anti‐CD56 (BV570, Biolegend, San Diego, CA, US), anti‐CD16 (PerCP‐Cy5.5, Biolegend, San Diego, CA, USA), anti‐HLA‐DR (APC, BD, Franklin Lakes, NJ, USA), anti‐CD11b (BV786, Biolegend, San Diego, CA, USA), anti‐CD11c (PE‐Cy5, BD, Franklin Lakes, NJ, USA), and anti‐CD14 (FITC, BD Horizon, Franklin Lakes, NJ, USA). Both cell fractions were finally sorted using the FACSAria™ III cell sorter (BD Bioscience, Franklin Lakes, NJ, USA). Sorting was achieved with the following strategy, B cells – CD3^‐^ CD19^+^, T cells – CD3^+^ CD19^‐^, NK cells – CD3^‐^ CD19^‐^ CD16^+^ CD56^‐^ or CD3^‐^ CD19^‐^ CD16^+^ CD56^+^ or CD3^‐^ CD19^‐^ CD16^‐^ CD56^+^, DCs – CD3^‐^ CD19^‐^ CD16^‐^ CD56^‐^ CD14^‐^ CD11b^‐^ CD11c^+^ HLA‐DR^+^, PMN – CD66b^+^ CD16^+^.

### 2.3 Quantitative real‐time PCR

For quantitative real‐time PCR assay (qRT‐PCR), cells were lysed with PureZOL Reagent (Bio‐Rad, Hercules, CA, USA) and total RNA was extracted using the Direct‐zol RNA Miniprep (Zymo Research, Irvine, CA, USA). RNA was retrotranscribed into cDNA using the High‐Capacity cDNA Reverse Transcription Kit (Applied Biosystems, Foster City, CA, USA), and transcript quantification was performed following the recommended protocols for Fast SYBR Green Master Mix (Applied Biosystems, Foster City, CA, USA) using specific primers (*GAPDH*, F ‐ 5′‐AGA TCA TCA GCA ATG CCT C‐3′, R – 5′‐ATG GCA TGG ACT GGG TCA‐3′; *BACTIN*, F ‐ 5′‐CCC AAG GCC AAC CGC GAG AAG AT‐3′, R – 5′‐ GTC CCG GCC AGC CAG GTC CAG‐3′; *B2M*, F ‐ 5′‐GCT CCG TGG CCT TAG CTG T‐3′, R – 5′‐ACG TGA GTA AAC CTG AAT CTT TGG A‐3′; *MS4A1*, F ‐ 5′‐CAC CCA TCT GTG TGA CTG TGT G‐3′, R – 5′‐AGT TTT TCT CCG TTG CTG CC‐3′; *MS4A3*, F ‐ 5′‐CAG AGT CAC CGG ACC TAT GC‐3′, R – 5′‐CAG TTT GCA TTG CAC CAC AT‐3′; *MS4A4A*, F ‐ 5′‐CTG GGA AAC ATG GCT GTC ATA‐3′, R – 5′‐CTC ATC AGG GCA GTC AGA ATC‐3′; *MS4A4E*, F ‐ 5′‐TTC TGA TTG CCT TGA TGA GC‐3′, R – 5′‐TAA GGA TAC ATC ACT GAC CC‐3′; *MS4A6A*, F ‐ 5′‐CTG GTG GGT TTC ATT ATC CT‐3′, R – 5′‐CAG ACT GGC TTT GGC TGT AT‐3′; *MS4A7*, F ‐ 5′‐CAC CAA AGG GCA TCA CTA TCC‐3′, R – 5′‐GAA ATC AAC AGG CAA CAC AGG‐3′; *MS4A14*, F ‐ 5′‐TCT TGC CTT CGG ATG TTA CTC A‐3′, R – 5′‐TGG TTG GGA GAC TAA AGG ACT C‐3′; *CLEC7A* *, F ‐ 5'‐CCT GGG TA CCA TGG CTA TTT‐3', R ‐ 5'‐GGG TTG ACT GTG GTT CTC TT‐3'; TREM2, F ‐ 5'‐CTG CTC ATC TTA CTC TTT GTC AC‐3'; R ‐ 5'‐CAG TGC TTC ATG GAG TCA TAG G‐3'*). Quantitative PCR was performed using the QuantStudio™ 7 Flex Real‐Time PCR System (Applied Biosystems™, Foster City, CA, USA).

### 2.4 RA patient's samples

RA patients underwent synovial sampling of an actively inflamed joint using ultrasound‐guided needle biopsy (as previously described^37^). All patients fulfilled the American College of Rheumatology/European League Against Rheumatism 2010 RA classification criteria,^38^ and all procedures were performed following written informed consent and were approved by the hospital's ethics committee (Rec number 10/H0801/47). At the time of biopsy, all patients were previously exposed to conventional synthetic disease modifying anti‐rheumatic drugs (csDMARD) but were naïve to biologic DMARD and some patients were concomitantly treated with steroids. Synovial tissue samples were fixed in 4% formaldehyde and embedded in paraffin for histological analysis.

### 2.5 Immunohistochemistry

Three‐micrometer‐thick formalin‐fixed, paraffin‐embedded sections of prototypic human lung and colon, synovium, and testis were deparaffinized and incubated with 3% hydrogen peroxide solution for 10 min at room temperature (RT) to quench endogenous peroxidase after Ag retrieval. Sections were then blocked with Background Sniper (Biocare Medicals, Pacheco, CA, USA; colon, lung, and testis) or Blocking solution (synovium; Dako, Santa Clara, CA, USA) for 20 min at RT and incubated with rabbit polyclonal antibodies targeting human MS4A3 (HPA019210, dilution 1:100; Sigma, St Louis, MO, USA), MS4A4A (HPA029323, dilution 1:200; Sigma), MS4A4E (HPA040075, dilution 1:50; Sigma), MS4A6A (HPA011391, dilution 1:50; Sigma), MS4A7 (HPA017418, dilution 1:50; Sigma), or MS4A14 (orb157902, dilution 1:200; Biorbyt, Cambridge, GB) for 1 h at RT or overnight at 4°C (MS4A7, synovium). Tissue sections were then incubated with MACH1 HRP‐polymer for 30 min at RT (colon, lung, and testis; Biocare Medical, Pacheco, CA, USA) or EnVision HRP System (synovium; Dako, Santa Clara, CA, USA). The chromogen reaction was developed using 3,3′‐Diaminobenzidine tetrahydrochloride (DAB; Biocare Medical, Pacheco, CA, US, or Dako, Santa Clara, CA, USA) and the sections were counterstained with hematoxylin. Immunostained sections were acquired using the Olympus BX51 Microscope equipped with a XC50 Color Camera and cell^F software (colon, lung, and testis; Olympus, Tokyo, Japan) or digitally scanned using Nanozoomer S210 (synovium; Hamamatsu Photonics, Hamamatsu, Japan). The percentage of MS4A7‐positive cells within the synovial tissue was calculated by quantitative digital image analyses using QuPath software.^39^

### 2.6 Immunohistochemistry of COVID‐19 lung samples

Five‐micrometer‐thick paraffin‐embedded archival lung tissue from deceased patients with SARS‐CoV‐2 positive pneumonia was stained on the Bond III automated staining platform (Leica, Wetzlar, DE). After deparaffinization and Ag retrieval, slides were incubated with Bond Peroxide Block for 5 min. Samples were then incubated with the primary monoclonal mouse anti‐human CD68 (clone KP1, dilution 1:7000; Dako, Santa Clara, CA, USA) and with the primary monoclonal MS4A4A (HPA029323, dilution 1:500; Sigma), followed by signal amplification and visualization using the Leica Bond Polymer Refine Detection Kit (Leica, Wetzlar, DE). Operating parameters for application of the detection system reagents were followed as suggested and incorporated into the software by Leica Biosystems. Finally, the slides were counterstained with hematoxylin, dehydrated and cover‐slipped.

### 2.7 Immunofluorescence

Double fluorescent labelling of MS4A7 (HPA017418, Sigma, dilution 1:50) and CD68 (M0814, dilution 1:50; Dako) was performed on synovial sections by multiplex immunofluorescence staining using a tyramide signal amplification protocol (Invitrogen, Thermo Fisher Scientific). All the slides were counterstained with DAPI (Invitrogen, Thermo Fisher Scientific) and digitally scanned using Nanozoomer S60 (Hamamatsu Photonics).

### 2.8 Gene expression analysis of PBMC RNA‐seq datasets

Bulk RNA‐seq data relative to normal PBMC cell populations were retrieved from various available sources, each of them constituted of a variety of sorted cell types. In particular, 3 comprehensive and widely used collections of transcriptional data were chosen to represent blood cells, being the most complete repositories, whose derived signatures are included in immune cells annotation pipelines. Among them, Haemopedia collection, deposited under the Gene Expression Omnibus (GEO) accession code GSE115736^40^ and Monaco G. dataset, deposited under the GEO ID GSE107011^41^ were publicly available and downloaded with *SRAToolkit* (http://ncbi.github.io/sra‐tools/). RNA‐Seq data relative to PBMC cells in normal condition, produced within the large data collection belonging to the Blueprint consortium under the accession ID EGAS00001000284 and EGAS00001000327,^42^, ^43^, ^44^ were retrieved upon previous request from The European Genome‐Phenome Archive (EGA) through the *pyega3* application. The 3 cohorts were analyzed separately (based on each individual cell classifications) and in a second phase joined in a normalized, merged dataset, using a broader cell lineage assignment definition.

For each dataset, raw reads were aligned and quantified using *STAR* version 2.7.b^45^ on the GRCh38 genome guided by GENCODE annotation (version 33). Gene summarized counts, for the individual and merged sets respectively, were processed in R software, after filtering out genes whose expression was minor than 2 reads; then counts were normalized with the R package *DESeq2*.^46^ Conversions between gene identifiers were performed with the “*org.Hs.eg.db*” library (Carlson, M. 2019. org.Hs.eg.db: Genome wide annotation for Human). Plots were rendered with the R library “ggplot2.” Correlations among family member genes were computed and represented with the “*corrplot”* library.

### 2.9 Epigenetic control of MS4A family

Chromatin immunoprecipitation sequencing data (ChIP‐Seq) were obtained from the Blueprint collection of experiments and derived through DCC portal (http://dcc.blueprint‐epigenome.eu/#/home). Bigwig tracks corresponding to histone modifications bindings were retrieved and imported into Integrative Genomics Viewer (IGV).^47^

Data relative to ChIP‐Seq of PU.1, RXR, and PPARG in THP‐1 cells were retrieved from the work of Pott et al., available under the accession ID GSE25426.^48^ Annotated processed peaks were retrieved as deposited by respective authors, converted to bed format and lifted to genome assembly release hg38 with UCSC LiftOver tool. ChIP‐seq experiments of PU.1 in RAMOS cell line were obtained from Senigl et al., accession ID GSE139810.^49^ Available bigwig files corresponding to peaks track were obtained as processed by the authors and lifted to genome assembly release hg38 with “Crossmap.py” converter.^50^ Converted tracks were imported to IGV for visualization and comparison.

### 2.10 mRNA isoform expression analysis of PBMC RNA‐seq datasets

Isoform quantification was performed for the PBMC cohorts starting from raw reads using *kallisto* ^51^ based on the GENCODE transcript annotation version 23. Normalized counts were compared and depicted in R environment. Heatmaps representing normalized tpm for a subset of MS4A family member genes were generated with the R package *“Pheatmap*.”

### 2.11 Gene expression analysis of RNA‐seq normal human tissue dataset

Publicly available RNA‐seq dataset relative to various tissues in physiological conditions was retrieved from the National Genomics Data Center BIGD database with accession code ID: PRJCA000751.^52^ The dataset includes for each organ, samples processed with 3 different RNA extraction protocols, polyadenylated selection, total RNA, and RNAse‐processed RNA, allowing the detection of all types of transcripts (respectively mRNA, complete set of coding and noncoding RNA, and transcripts with stable architectures). Paired end files of raw reads were aligned, quantified, and normalized following the same pipeline described for PBMC datasets.

### 2.12 RNA sequencing of COVID‐19 peripheral neutrophils and gene expression analysis

The study on COVID19 patients was approved by the local Ethical Committee (authorization 233/20). This study was conducted on patients admitted to Humanitas Clinical and Research Center (Rozzano, Milan, Italy) between March 4 and May 9 2020 with a laboratory‐confirmed diagnosis of COVID‐19. None of the patients were treated with corticosteroids prior to the blood withdrawal. mRNA from neutrophils isolated from whole blood by negative selection using MACSxpress^®^ Whole Blood Neutrophil Isolation Kit (Miltenyi Biotec, Bergisch Gladbach, DE) was purified with the Direct‐zol RNA Microprep or Miniprep Kits (Zymo Research, Irvine, CA, USA), according to manufacturer's instructions. Total‐RNA‐sequencing library preparation was performed starting from 1 ng of total‐RNA with the SMART‐Seq Stranded Kit (Clontech‐Takara, Mountain View, CA, USA). Libraries obtained were qualitatively assessed by using TapeStation 4200 (Agilent, Santa Clara, CA, USA) and quantified by Qubit Fluorimeter (Thermofisher, Waltham, MA, USA). Afterwards, they were multiplexed in equimolar pools and sequenced on a NextSeq‐550 Illumina Platform generating at least 80 million 75 bp‐PE reads per sample. Raw RNA‐Seq data of human peripheral neutrophils derived from 3 healthy donors were retrieved from GEO under the series GSE163533.

Raw files included in all the experiments were aligned and quantified with STAR (version 2.6.1) on the GRCh38 genome guided by GENCODE annotation (version 33). Gene summarized counts were processed in R software, genes whose expression was minor than 2 reads were removed while the remaining portion was “vs. normalized” with the R package DESeq2. Plots were rendered with the R library “ggplot2.”

### 2.13 COVID‐19 bronchoalveolar lavage fluid (BALF) and COVID‐19 peripheral blood single cell RNA‐Seq data analysis

Data relative to scRNA‐Seq of COVID‐19 individuals was retrieved from public platforms: COVID‐19 BALF experiments were derived from the COVID‐19 Cell Atlas with the ID “Bronchoalveolar lavage fluid VIB U‐Gent.”^53^ Data relative to COVID‐19 PBMC were obtained from the platform FASTGenomics under the ID “*Schulte‐Schrepping 2020 COVID19 10x PBMC”* and *“Schulte‐Schrepping 2020 COVID19 Rhapsody PBMC*.”^54^ Data were retrieved as provided by the platforms and visualized through CellXGene interactive explorer.

### 2.14 Statistical analysis

Results were expressed as median ± IQR (interquartile range) from multiple independent experiments and donors, as indicated in figures legends. Kruskal‐Wallis (>2 groups) and Mann‐Whitney (2 groups) nonparametric tests were performed using Prism (GraphPad), after ROUT test was applied for outlier determination. Results were considered significant if *P *≤ 0.05.

## 3 RESULTS

### 3.1 Analysis of *MS4A* gene expression in human hematopoietic cells

**FIGURE 2:**
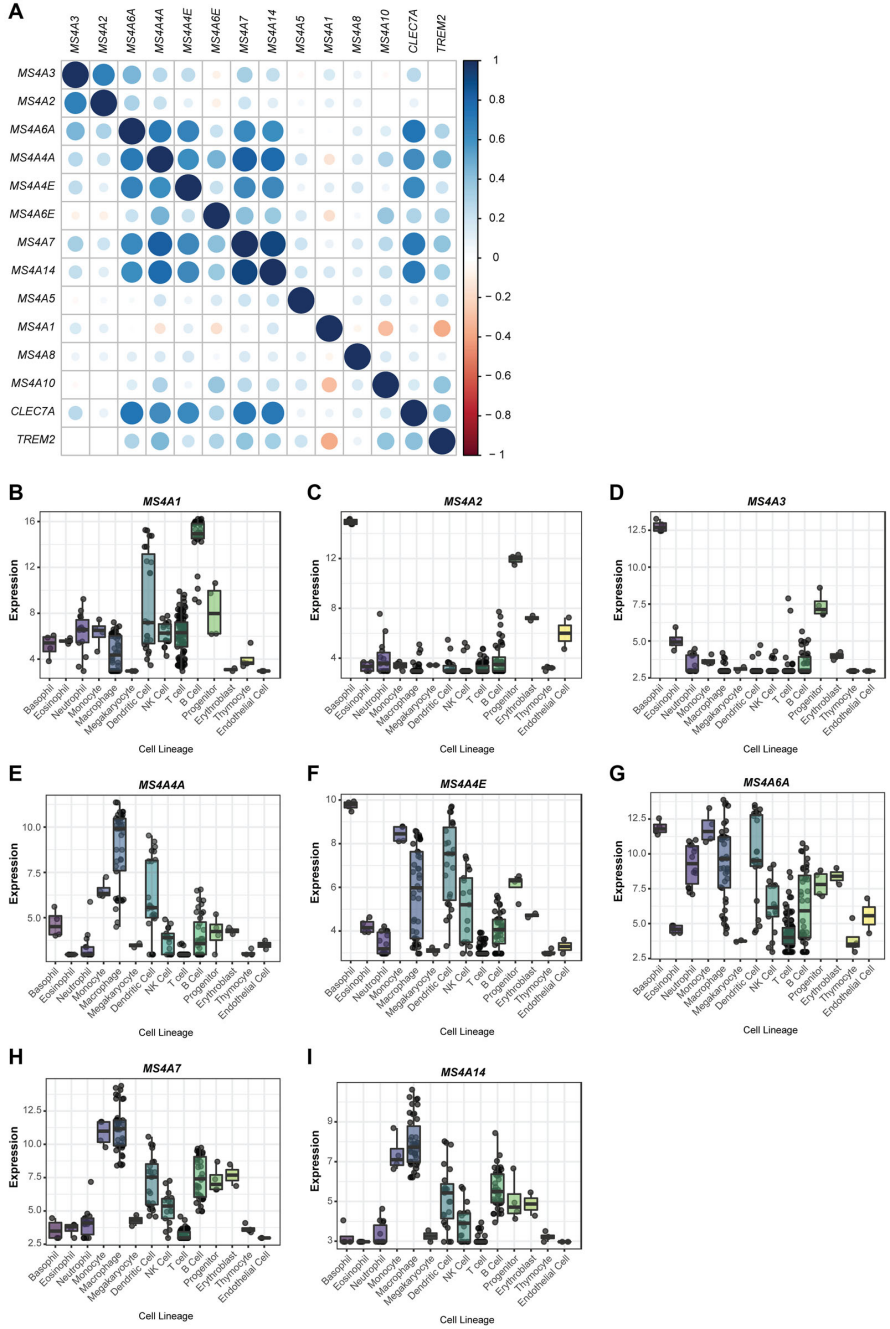
**Expression of MS4A family genes in public data sets of human cells. (A)** Correlation of the expression of 13 *MS4A* genes in human leukocytes. The circle size is proportional to the absolute correlation value. Blue – positive correlation; Red – negative correlation. Expression of the *MS4A1* **(B)**, *MS4A2* **(C)**, *MS4A3* **(D)**, *MS4A4A* **(E)**, *MS4A4E* **(F)**, *MS4A6A* **(G)**, *MS4A7* **(H)**, and *MS4A14* **(I)** genes in human leukocyte lineages and other cells. **(B‐I)** Expression is represented as Log2 of the normalized expression value. RNA‐seq data was retrieved from *Choi J. et al. Nucleic Acids Res (2019)* ^40^ (GSE115736), *Monaco G. et al. Cell Reports (2019)* ^41^ (GSE107011) and *Chen L. et al. Cell (2016)* ^42^ (EGAS00001000284 and EGAS00001000327)

To characterize the expression of selected MS4A family members in human leukocytes, we analyzed 3 public RNA sequencing (RNA‐seq) datasets of hematopoietic cells [GSE115736,^40^ GSE107011,^41^ EGAS00001000284, and EGAS00001000327^42^, ^43^, ^44^, ^55^]. From the 16 genes of the *MS4A* family located in Chr 11q12 (Fig. 1), only 13 were detected in the 3 RNA‐seq datasets analyzed. First, we evaluated the correlation between the expression of these genes in hematopoietic cells and we observed 2 positive correlation clusters. One cluster included *MS4A2* and *MS4A3*, while a second one comprised *MS4A4A*, *MS4A4E*, *MS4A6A*, *MS4A6E*, *MS4A7*, and *MS4A14* (Fig. 2A), suggesting a similar expression pattern across the different leukocyte populations. Interestingly, *TREM2* and *CLEC7A* genes, which were previously reported as molecular partners of MS4A4A,^9^, ^27^ also correlated with *MS4A4A*, *MS4A6A*, *MS4A7*, *MS4A14*, and *MS4A6E* or *MS4A4E*, respectively (Fig. 2A). Of note, and in accordance with previously reported data,^56^, ^57^ we confirmed that *CLEC7A* and *TREM2* were mainly expressed by myeloid cells (*data not shown*). The individual analysis of the 3 datasets is reported in Supplementary Fig. S1‐3. When assessing the expression of each *MS4A* gene in different hematopoietic cell populations, we observed that *MS4A1*, besides being expressed mainly by B cells, as already reported,^4^, ^11^ was also surprisingly expressed by a fraction of cells belonging to the dendritic cell (DC) lineage (Fig. 2B). A detailed analysis of the 3 datasets revealed that only in one of them *MS4A1* was expressed by DCs (Supplementary Fig. S3A). Both *MS4A2* and *MS4A3* were expressed by basophils and CD34^+^ hematopoietic progenitor cells (Fig. 2C and D, respectively), confirming the positive correlation between the expression of the 2 genes. *MS4A4A* was mostly detected in Mϕs, and to a lower extent in a fraction of DCs and monocytes (Fig 2E). *MS4A4E* was mainly expressed by basophils and monocytes, and was also detected in DCs, Mϕs, NK cells, and progenitor cells (Fig 2F). *MS4A6A* was broadly expressed, presenting the highest expression levels in the myeloid compartment, including Mϕs, DCs, basophils, and neutrophils (Fig. 2G). Importantly, the expression of *MS4A6A* by the monocyte lineage was restricted to the classical monocyte subset (Supplementary Fig. S1‐3, panel F). *MS4A7* was mainly detected in monocytes and Mϕs, with a lower expression in B cells, DCs, and progenitor cells (Fig. 2H). Of note, the expression of this gene by monocytes, Mϕs, and B cells was more consistent between datasets, than the expression in DCs or progenitor cells (Supplementary Fig. S1‐3, panel G). *MS4A14* (Fig. 2I) expression pattern was very similar to *MS4A7* (Fig. 2H), further confirming the positive correlation between the 2 genes (Fig. 2A).

These data showed that *MS4A2*, *MS4A3*, *MS4A4A*, *MS4A6A*, *MS4A7*, and *MS4A14* genes were mainly, although not exclusively, expressed by cells belonging to the myeloid compartment, whereas the expression of *MS4A1* and *MS4A4E* was detected in the lymphoid and myeloid compartments. Other genes were poorly expressed or even not detected in hematopoietic cells (*data not shown*).

**FIGURE 3:**
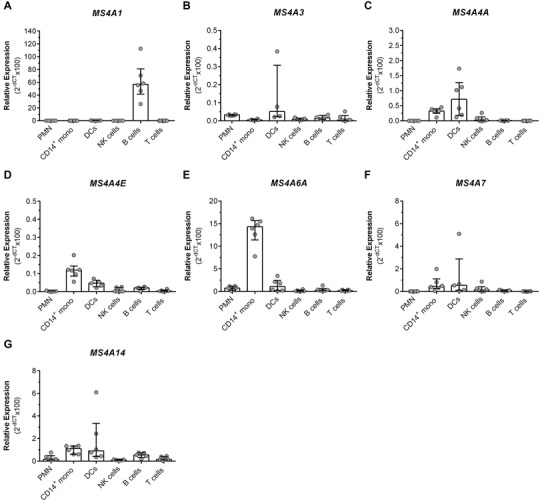
***MS4A* gene expression in human leukocytes**. mRNA expression of *MS4A1* **(A)**, *MS4A3* **(B)**, *MS4A4A* **(C)**, *MS4A4E* **(D)**, *MS4A6A* **(E)**, *MS4A7* **(F)**, and *MS4A14* **(G)** genes by human blood leukocytes. Five to 6 independent biological replicates are presented for each gene and cell type. PMN, CD14^+^ monocytes, DCs, NK cells, B cells, and T cells were sorted from blood based on their protein membrane expression (See Methods section). Data are represented by each biological replicate (white symbols) and median (bars) as relative expression to 3 housekeeping genes (*GAPDH*, *bACTIN*, and *b2M*)

In order to validate the bioinformatics analysis of RNA‐seq public databases, we sorted different leukocyte populations from freshly collected human blood and evaluated the expression of *MS4A1*, *MS4A3*, *MS4A4A MS4A4E*, *MS4A6A*, *MS4A7*, and *MS4A14* by qRT‐PCR. We confirmed the expression of *MS4A1* in CD3^‐^ CD19^+^ B cells (Fig. 3A), while the expression of most of the *MS4A* genes tested was very low and difficult to be associated with a particular blood leukocyte subset (Fig. 3B, C, D, F, G). Importantly, we found that the expression of *MS4A6A* was high in CD14^+^ monocytes (Fig. 3E).

### 3.2 *MS4A* expression during monocyte‐to‐Mϕ differentiation

**FIGURE 4:**
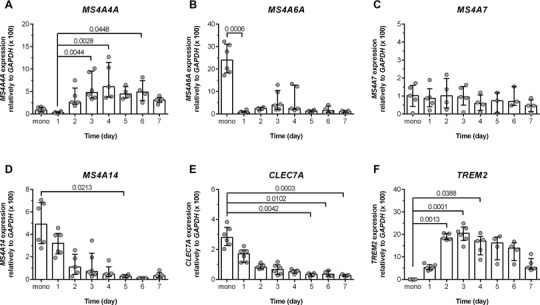
**Expression of *MS4A* genes during in vitro monocyte to M**ϕ **differentiation**. mRNA expression of *MS4A4A* **(A)**, *MS4A6A* **(B)**, *MS4A7* **(C)**, and *MS4A14* **(D)** genes during in vitro M‐CSF‐dependent monocyte to Mϕ differentiation. **(A‐D)** Data are presented as relative expression to *GAPDH*. Results are shown as median ± IQR, and each symbol represents a different biological replicate. *N* = 4–6 (per group). Statistical analysis was performed by Kruskal‐Wallis test with a Dunn's multiple comparison test; *P *≤ 0.05 was considered significantxs

In silico analysis of RNA‐seq data suggested that *MS4A3, MS4A4A, MS4A4E, MS4A6A, MS4A7*, and *MS4A14* are expressed by myeloid cells, in particular cells belonging to the monocyte‐Mϕ lineage. We have previously reported that MS4A4A expression is acquired during Mϕ differentiation, both at the RNA and protein level.^9^, ^23^ Therefore, we evaluated the expression of other *MS4A* genes during in vitro monocyte‐to‐Mϕ differentiation driven by M‐CSF. While *MS4A3* and *MS4A4E* were essentially undetectable (*data not shown*), we confirmed the increase in *MS4A4A* expression during monocyte‐to‐Mϕ differentiation (Fig. 4A). In contrast, *MS4A6A* (Fig. 4B) and *MS4A14* (Fig. 4D) were expressed at higher level in monocytes [median (IQR) = 24.02 (18.31‐31.10); 4.91 (3.18‐6.82), respectively] compared to *MS4A4A* [0.90 (0.52‐1.52), *P* = 0.002 (Mann‐Whitney)], and their expression decreased during differentiation. Finally, *MS4A7* expression was not modulated during monocyte to Mϕ differentiation in vitro (Fig. 4C). Of note, we have observed that while *CLEC7A* expression decreased during Mϕ differentiation (Fig. 4E), *TREM2* followed a similar expression pattern to *MS4A4A* (Fig. 4F).

We previously reported that MS4A6A could interact with MS4A4A.^9^ Although its mRNA expression decreased during monocyte to Mϕ differentiation (Fig. 4B), MS4A6A protein was still detectable in the membrane of mature Mϕs^9^ and potentially able to interact with MS4A4A.

These results indicate that *MS4A4A* and *MS4A6A* are differentially expressed in monocytes and Mϕs (Fig. 4A‐B). MS4A4A protein has previously been shown to be present in Mϕs.^9^ In contrast, we could not confirm the *MS4A6A* mRNA expression in monocytes at the protein level by flow cytometry analysis (*data not shown*).

We then assessed the expression of mRNA isoforms. For most of the analyzed genes, there was no differential distribution of their mRNA isoforms among cell types (Supplementary Fig. S4). In contrast, we observed a differential *MS4A6A* isoform usage between monocytes and Mϕs from cord‐blood origin (Supplementary Fig. S4). Additionally, we noticed that the majority of *MS4A4A* and *MS4A7* isoforms clustered together, indicating that their transcriptional profile across cell populations is similar, and that they do not undergo a differential exon usage in different cellular environments. Conversely, *MS4A14* exhibited different isoforms in monocytes/Mϕs compared to alternatively activated Mϕs (Supplementary Fig. S4).

### 3.3 Transcriptional regulation of *MS4A* genes during Mϕ activation

**FIGURE 5:**
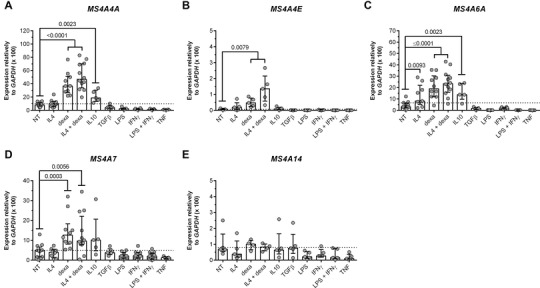
**Regulation of *MS4A* gene expression by glucocorticoid hormones**. Transcript expression of *MS4A4A* **(A)**, *MS4A4E* **(B)**, *MS4A6A* **(C)**, *MS4A7* **(D)**, and *MS4A14* **(E)** genes by monocyte‐derived Mϕ treated or not (NT) for 18 h as indicated. Data are presented as relative expression to *GAPDH*. Results are shown as median ± IQR, and each symbol represents a different biological replicate. N = 5‐10 (per group). Statistical analysis was performed using Mann‐Whitney test compared to non‐treated cells (NT); *P *≤ 0.05 was considered significant

Dexamethasone and IL‐4 have previously been shown to enhance expression of MS4A4A.^9^, ^58^ It was therefore important to assess how regulators of myeloid cell function affect members of the MS4A family. The expression of *MS4A4A* (Fig. 5A), *MS4A4E* (Fig. 5B), *MS4A6A* (Fig. 5C), and *MS4A7* (Fig. 5D) was up‐regulated by dexamethasone alone and in combination with IL‐4. It should be noted that *MS4A4E* expression was generally very low in Mϕs. Moreover, *MS4A4A* (Fig 5A) and *MS4A6A* (Fig. 5C) were induced by IL‐10 treatment. In contrast, the expression of *MS4A14* was unaffected (Fig. 5E). Thus, glucocorticoids, prototypic anti‐inflammatory agents,^24^ augment the expression of a subset of MS4A family members in mononuclear phagocytes.

**FIGURE 6:**
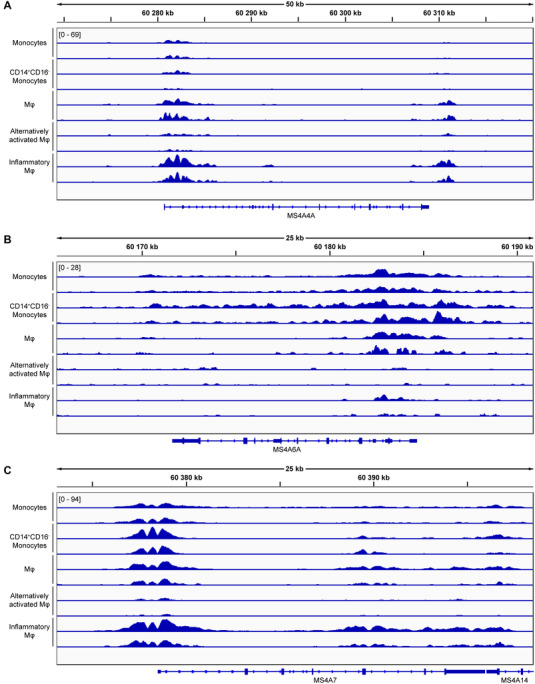
**Graphical representation of H3K27ac histone mark peaks in human cells**. Representation of ChIP‐seq data retrieved from Blueprint collection of experiments and derived through DCC portal (http://dcc.blueprint‐epigenome.eu/#/home) showing H3K27ac histone mark peaks in **(A)** *MS4A4A*, **(B)** *MS4A6A*, and **(C)** *MS4A7* genes. Data of 2 replicates for each cell type are depicted in the figure

Nevertheless, the transcriptional regulation at the molecular level of MS4A family members has been poorly studied so far. Thus, to further explore the regulation of these genes, we have analyzed H3K27ac ChIP‐Seq data retrieved from DCC portal (http://dcc.blueprint‐epigenome.eu/#/experiments). This histone mark is usually associated with induction of gene expression. By comparing the peaks of H3K27ac in monocytes, CD14^+^CD16^‐^ classical monocytes, Mϕs, alternatively activated Mϕs (IL‐13 and Rosiglitazone treatment), and inflammatory Mϕs (LPS treatment), we observed an enrichment of this histone mark in *MS4A6A* genomic region in monocytes and CD14^+^CD16^‐^ classical monocytes (Fig. 6B). *MS4A4A* binding signal was increased in Mϕs and *MS4A7* H3K27ac peaks did not change between monocytes and Mϕs (Fig. 6A and C), confirming our gene expression data. PU.1 was suggested to act as a “global genome organizer” in Mϕs^59^ and to be involved in the alternative activation of Mϕs.^60^ To test the possible transcriptional activation of these genes by PU.1 binding, we analyzed ChIP‐Seq data of PU.1 in THP‐1 cells differentiated with PMA (phorbol 12‐myristate 13‐acetate), representing a Mϕ‐like cell model (GSE25426), and Ramos cells, a B cell line derived from Burkitt lymphoma (GSE139810).^48^, ^49^ We found that in THP‐1 cells, PU.1 was bound to the promoter region of *MS4A4A*, *MS4A7*, and *MS4A14*, but not of *MS4A1* (Supplementary Fig. S5). Conversely, in Ramos cells, PU.1 was mainly found in the promoter region of *MS4A1* (Supplementary Fig. S5). Together, these data are in line with the expression results (Fig. 2B, E, G‐I) and suggest for a potential involvement of PU.1 in the transcriptional regulation of *MS4A* genes.

### 3.4 MS4A gene expression in human tissues

**FIGURE 7:**
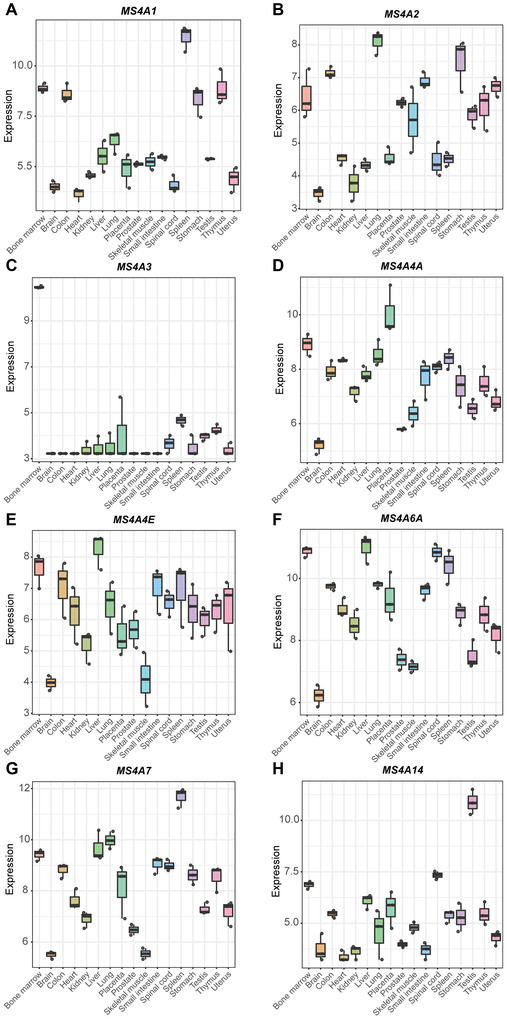
**Expression of *MS4A* genes in human tissues**. Expression of *MS4A1* **(A)**, *MS4A2* **(B)**, *MS4A3* **(C)**, *MS4A4A* **(D)**, *MS4A4E* **(E)**, *MS4A6A* **(F)**, *MS4A7* **(G)**, and *MS4A14* **(H)** in different human healthy tissues. RNA expression data were retrieved from Ji P. et al. *Cell Reports (2019)* ^52^ (https://bigd.big.ac.cn/gsa/browse/CRA000348). Data are represented as Log_2_ of the normalized expression value. Each dot represents 1 of 3 different RNA isolation methods for the same sample, including total RNA, poly(A) enrichment and RNase R treated RNA

To characterize the expression of selected *MS4A* genes (*MS4A1*, *MS4A2*, *MS4A3*, *MS4A4A*, *MS4A4E*, *MS4A6A*, *MS4A7*, and *MS4A14*) in human tissues, we investigated RNA‐seq data from a public dataset of healthy human tissues (CRA000348).^52^ In accordance with its expression by B cells, *MS4A1* was mostly found in B cell enriched tissues, such as primary lymphoid organs (thymus), secondary lymphoid organs (spleen), and bone marrow (Fig. 7A). *MS4A2* was expressed in several tissues, but its expression was higher in lung and gastrointestinal tract (Fig. 7B). Interestingly, while *MS4A3* expression was almost restricted to the bone marrow (Fig. 7C), *MS4A14* was mainly detected in the testis (Fig. 7H), and a lower expression was observed in several other tissues. *MS4A4E* was broadly expressed, with its highest expression level being found in the liver (Fig. 7E). *MS4A4A*, *MS4A6A*, and *MS4A7* were also broadly expressed, and presented a similar expression profile across the different tissues (Fig. 7D, F, and G, respectively). The 3 genes were expressed in the bone marrow and secondary lymphoid organs as the spleen. Importantly, *MS4A4A*, *MS4A6A*, and *MS4A7* were expressed in Mϕ‐enriched organs, as the lung and liver, in agreement with their expression by cells of the monocyte/Mϕ lineage. Additionally, *MS4A4A* was also highly expressed in placenta (Fig. 7D) and *MS4A6A* in the spinal cord (Fig. 7F). Other members of the family, which are generally not expressed by hematopoietic cells, were expressed by specific tissues (Supplementary Fig. S6). For instance, *MS4A12* was mainly expressed in the colon (Supplementary Fig. S6E), while *MS4A5*, *MS4A6E*, and *MS4A13* were almost exclusively detected in testis (Supplementary Fig. S6A, B, and F, respectively).

**FIGURE 8:**
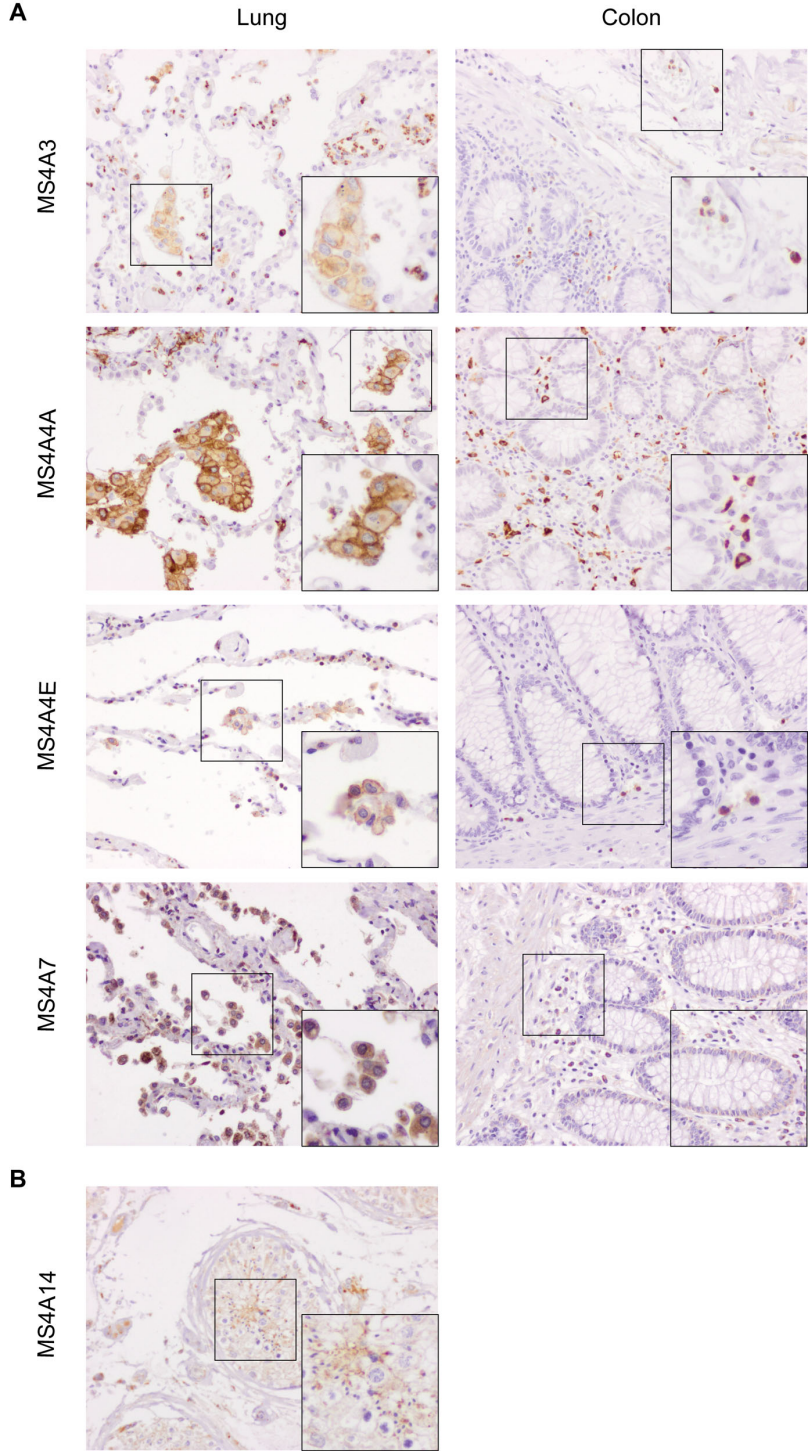
**Expression of MS4A proteins in human tissues**. Expression of MS4A3, MS4A4A, MS4A4E, MS4A7 **(A),** and MS4A14 **(B)** proteins in human tissue. Three micrometer slices of paraffin embedded lung and colon **(A)** or testis **(B)** tissue samples were stained with rabbit anti‐human MS4A3, MS4A4A, MS4A4E, MS4A7, and MS4A14 (brown) and counterstained with hematoxylin (blue). Figure (20×) and insert (40×) represent the expression in the normal/healthy portion of the sample

The expression of MS4A3, MS4A4A, MS4A4E, and MS4A7 proteins was then evaluated by immunohistochemistry in normal tissues. MS4A3 has been previously defined as a marker of early myeloid differentiation both in mouse and human, being expressed on the membrane of human blood monocytes and granulocytes.^19^ Our RNA‐seq analysis revealed that *MS4A3* was mainly expressed by basophils and progenitor cells (Fig. 2D). Interestingly, both in lung and colon tissue, MS4A3 was expressed in the cytoplasm of polymorphonuclear and mononuclear cells inside blood vessels, and at a lower extent in a subpopulation of Mϕ‐like cells (Fig. 8A). MS4A4A expression was restricted to the membrane of cells with a Mϕ morphology both in the lung and colon (Fig. 8A). Our RNA‐seq analysis indicated that *MS4A4E* was mostly expressed by myeloid cells (Fig. 2F). At the protein level, we observed the expression of MS4A4E in cells with a Mϕ morphology and in polymorphonuclear cells (Fig. 8A). Similarly to MS4A4A, MS4A7 was expressed by Mϕ‐like cells (Fig. 8A), but mostly in the cytoplasm and predominantly in the lung. The tissue RNA‐seq data showed that *MS4A14* was mainly expressed in the testis (Fig. 8H). When we analyzed the expression of this protein in samples of seminiferous tubules, we observed a localized staining in the most advanced stages of germinal cell development (Fig. 8B), namely spermatids and mature spermatozoa.

### 3.5 Expression of selected family members in rheumatoid arthritis and COVID‐19

**FIGURE 9:**
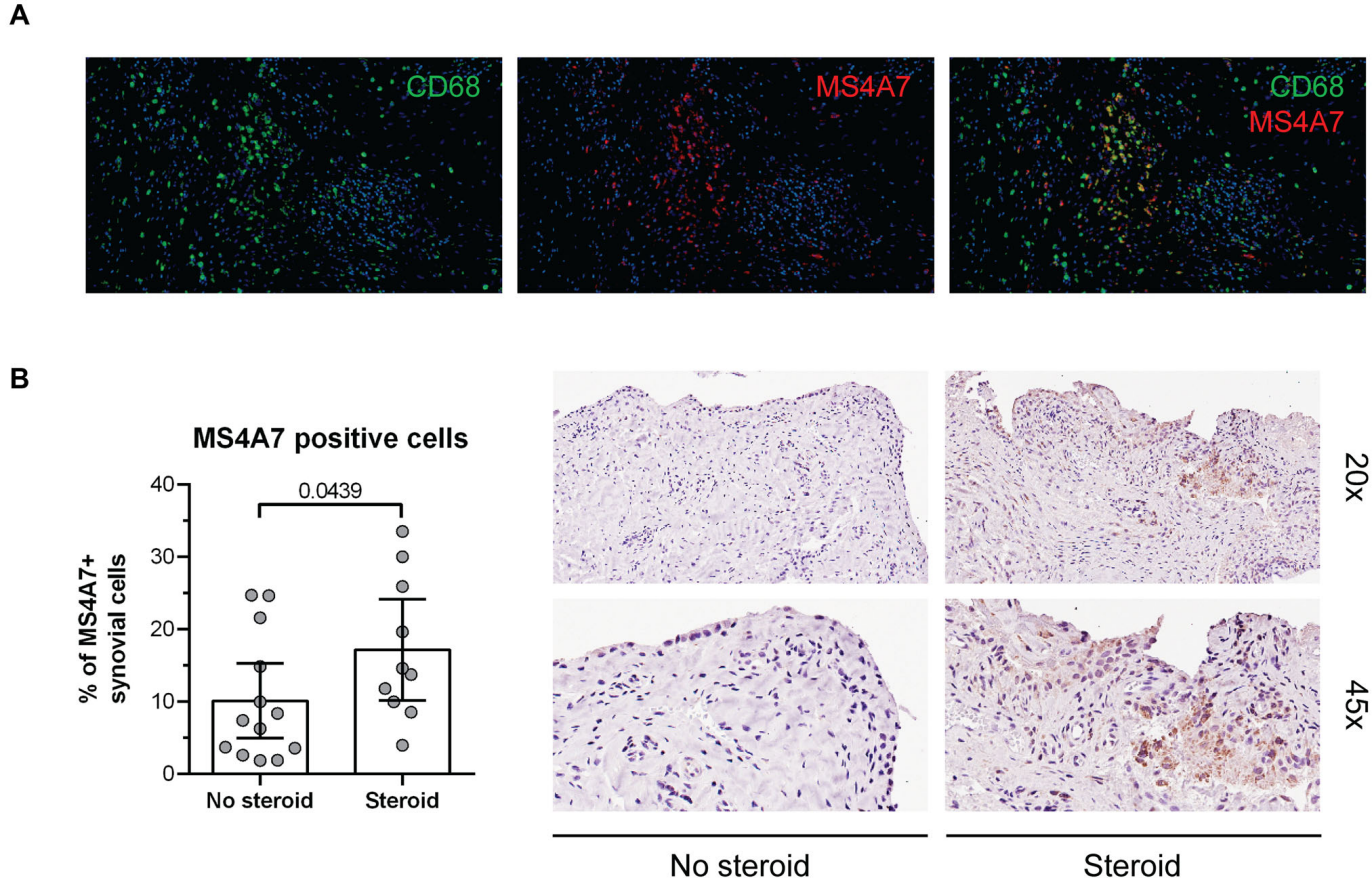
**Expression and glucocorticoid regulation of MS4A7 in RA synovia**. MS4A7 protein expression in synovial tissue of RA patients. **(A)** Representative image of CD68 and MS4A7 co‐expression in RA synovial tissue. **(B)** Quantification of the percentage of MS4A7 positive cells in synovial samples of RA patients treated or not with steroids. Representative images of MS4A7 expression in the tissue. Results are shown as median ± IQR, and each symbol represents a different biological replicate. *N* = 10–12. Statistical analysis by Mann–Whitney test; *P *≤ 0.05 was considered significant

To obtain preliminary indications as to the significance of selected MS4A family members in human pathology, we focused on diseases driven by a dysregulation of the myeloid compartment, such as rheumatoid arthritis (RA) and COVID‐19.^54^, ^61^, ^62^, ^63^ We have previously demonstrated that MS4A4A is highly expressed by CD68^+^ Mϕs in the synovial tissue of RA patients.^9^ Similarly, we observed here the co‐localization of MS4A7 and CD68 in the RA synovium (Fig. 9A). Moreover, the percentage of MS4A7 positive cells was significantly increased in the synovium of patients who received steroid treatment in conjunction to their conventional DMARD therapy compared to those who were only treated with DMARD (Fig. 9B). Thus, the analysis of MS4A7 expression in the synovial tissue of RA patients validated our in vitro findings and confirmed the in vivo regulation of this protein by

glucocorticoids.

**FIGURE 10:**
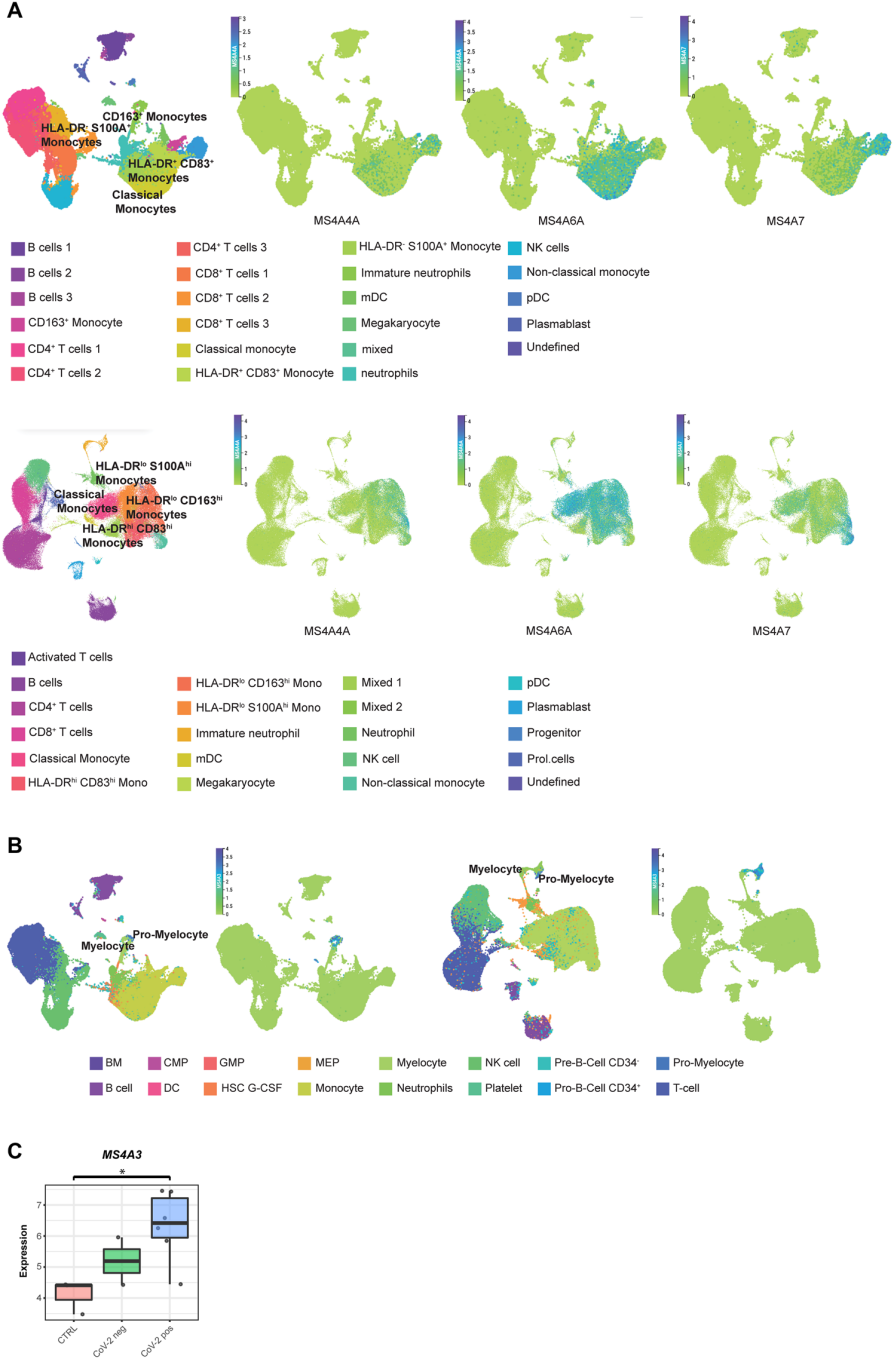
**Expression of *MS4A* genes in COVID‐19**. U‐map single cell RNA‐seq analysis of PBMC of COVID‐19 patients showing different cell population (multicolor panels) and *MS4A4A, MS4A6A* and *MS4A7* expression in those populations (green‐to‐blue panels) **(A)**, or different cell population (multicolor panels) and *MS4A3* expression in those populations (green‐to‐blue panels) **(B)**. **(A and B)** Represent single cell RNA‐seq data of 2 different cohorts retrieved from *Schulte‐Schrepping J. et al. Cell (2020)*.^54^ The data were retrieved from FASTGENOMICS platform under the ID “Schulte‐Schrepping_2020_COVID19_Rhapsody_PBMC” (**A** – upper panels, **B** – left panels) or “Schulte‐Schrepping_2020_COVID19_10x_PBMC” (**A** – bottom panels, **B** – right panels). Cell labels are assigned following “cluster_labels_res.0.4″ (defined by the authors) **(A)** or HPCA **(B)** annotation. **(C)** Expression of *MS4A3* by RNA‐seq analysis of sorted neutrophils from controls (CTRL), patients tested negative (CoV‐2 neg) or positive for SARS‐CoV‐2 (CoV‐2 pos). Expression data are represented as Log_2_ of the normalized expression value. *P* ≤ 0.05 was considered significant. *P ≤ 0.05, **P ≤ 0.005, ***P ≤ 0.001

**FIGURE 11:**
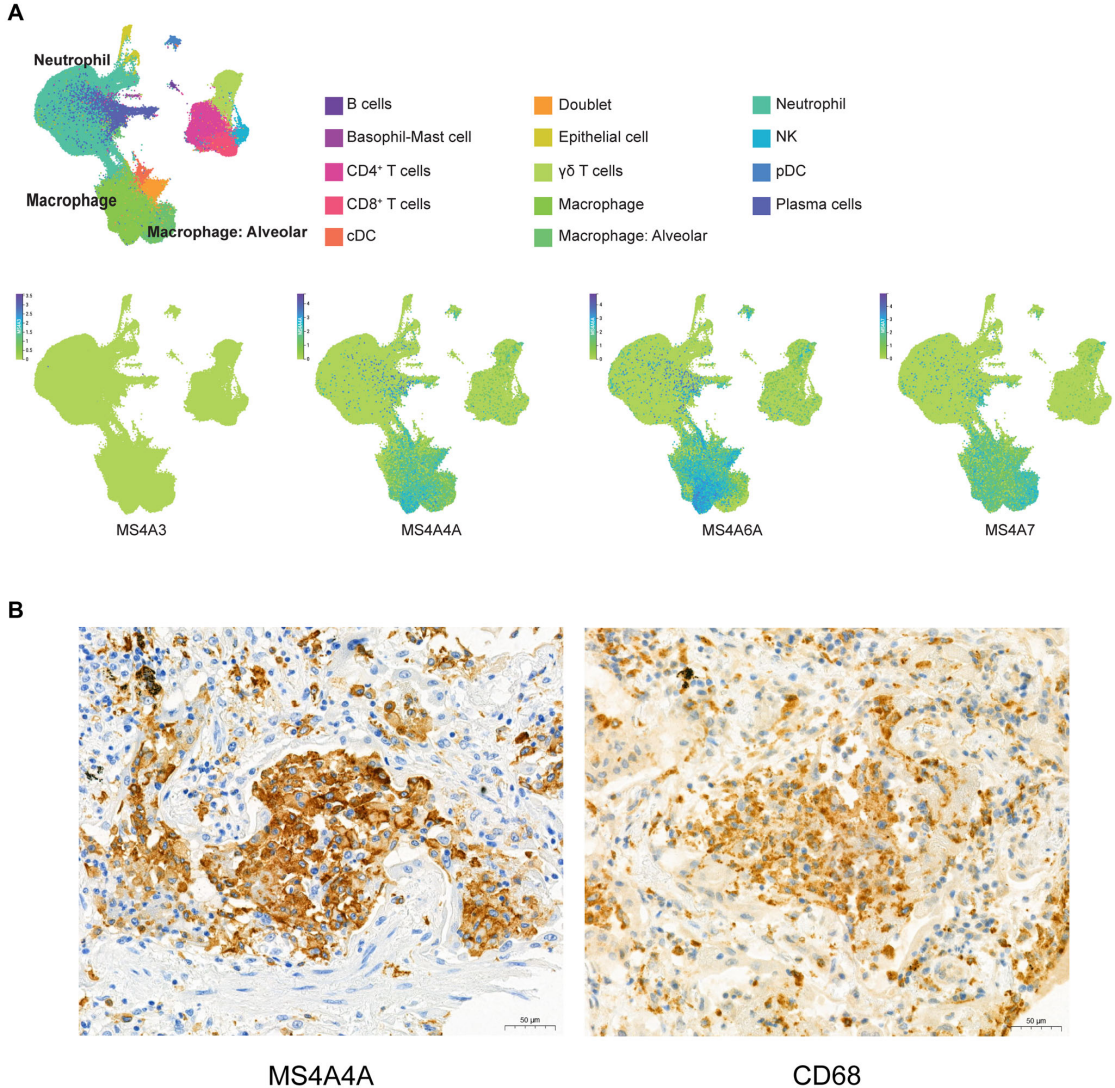
**Expression of *MS4A* genes in lung and BALF of COVID‐19 patients. (A)** U‐map single cell RNA‐seq analysis BALF of COVID‐19 patients showing different cell population (multicolor panel) and *MS4A3, MS4A4A, MS4A6A* or *MS4A7* expression in those populations (green‐to‐blue panels). The data were retrieved from the COVID‐19 Cell Atlas with the ID “Bronchoalveolar lavage fluid VIB‐UGent”, *Ballestar E. et al. (unpublished) (2021)*.^53^ **(B)** Expression of MS4A4A and CD68 in lung tissue of deceased patients with SARS‐CoV‐2 positive pneumonia. 5μm serial slices of paraffin embedded lung samples were stained with rabbit anti‐human MS4A4A or mouse anti‐human CD68 (brown) and counterstained with hematoxylin (blue)

An important dysregulation of the myeloid compartment was also described in SARS‐CoV‐2 infected patients.^54^, ^62^, ^63^, ^64^ We inspected the expression of members of the MS4A family in available datasets of single cell RNA‐seq derived from 2 cohorts of PBMC samples of COVID‐19 patients,^54^ and we confirmed that *MS4A4A*, *MS4A6A*, and *MS4A7* were expressed in monocytes (Fig. 10A). In particular, *MS4A4A* was transcribed by selective cell clusters described as “*HLA‐DR^low^, CD163^high^ monocytes*” and “*non‐classical monocytes*” (Fig. 10A). Similar patterns were observed for *MS4A7* transcription, which was additionally expressed in “*HLA‐DR^high^, CD83^high^ monocytes*” in both cohorts. In agreement with our previous observations in healthy samples, *MS4A6A* gene expression was found across the monocytic compartment, with prevalence in the “*classical monocyte*” population (Fig. 10A). In the same datasets, we investigated the abundance of *MS4A3* and confirmed the selective expression of this gene in “*pro‐myelocyte*” and “*myelocyte*” clusters, being considered a marker of these cellular entities (Fig. 10B). This observation is in line with the presence of dysfunctional and immature neutrophils in the circulation of COVID‐19 patients, as a result of emergency myelopoiesis.^54^, ^64^ Furthermore, we inspected in‐house performed bulk RNA‐seq data of circulating neutrophils of healthy, SARS‐CoV‐2 negative, and SARS‐CoV‐2 infected individuals, and we also found *MS4A3* to be increased in circulating neutrophils of COVID‐19 patients (Fig. 10C). Interestingly, by bioinformatics analysis of available single cell (sc)RNA‐seq data, *MS4A3* was not expressed in BALF of COVID‐19 patients (Fig. 11A). Similarly to what we observed in normal lung (Fig. 8A), immunohistochemistry on lung of deceased COVID‐19 patients confirmed an MS4A4A positivity restricted to CD68^+^ cells (Fig. 11B). The scRNA‐seq analysis supported these data, with *MS4A4A* being expressed by Mϕs or alveolar Mϕs in COVID‐19 BALF (Fig. 11A).

## 4 DISCUSSION

The human MS4A family comprises 18 genes, 16 organized as a cluster on Chr 11q12 (Fig. 1) and 2 on Chr 7q36.1.^1^, ^7^ Specific functions have been described for 3 members of the family (MS4A1/CD20; MS4A2; MS4A4A) and MS4A1/CD20 has proven to be an invaluable therapeutic target for the treatment of B cell malignancies and autoimmune diseases.^9^, ^11^, ^12^, ^13^, ^15^, ^17^, ^25^, ^26^ Thus, 15 members of the MS4A family belong to the “ignorome,” the druggable fraction of the genome with unknown function.^34^, ^35^, ^36^ The present study was designed to obtain a fingerprint of the expression of MS4A family members in humans with a focus on myelomonocytic cells, as a premise for search for function and, possibly, therapeutic targeting.

**FIGURE 12:**
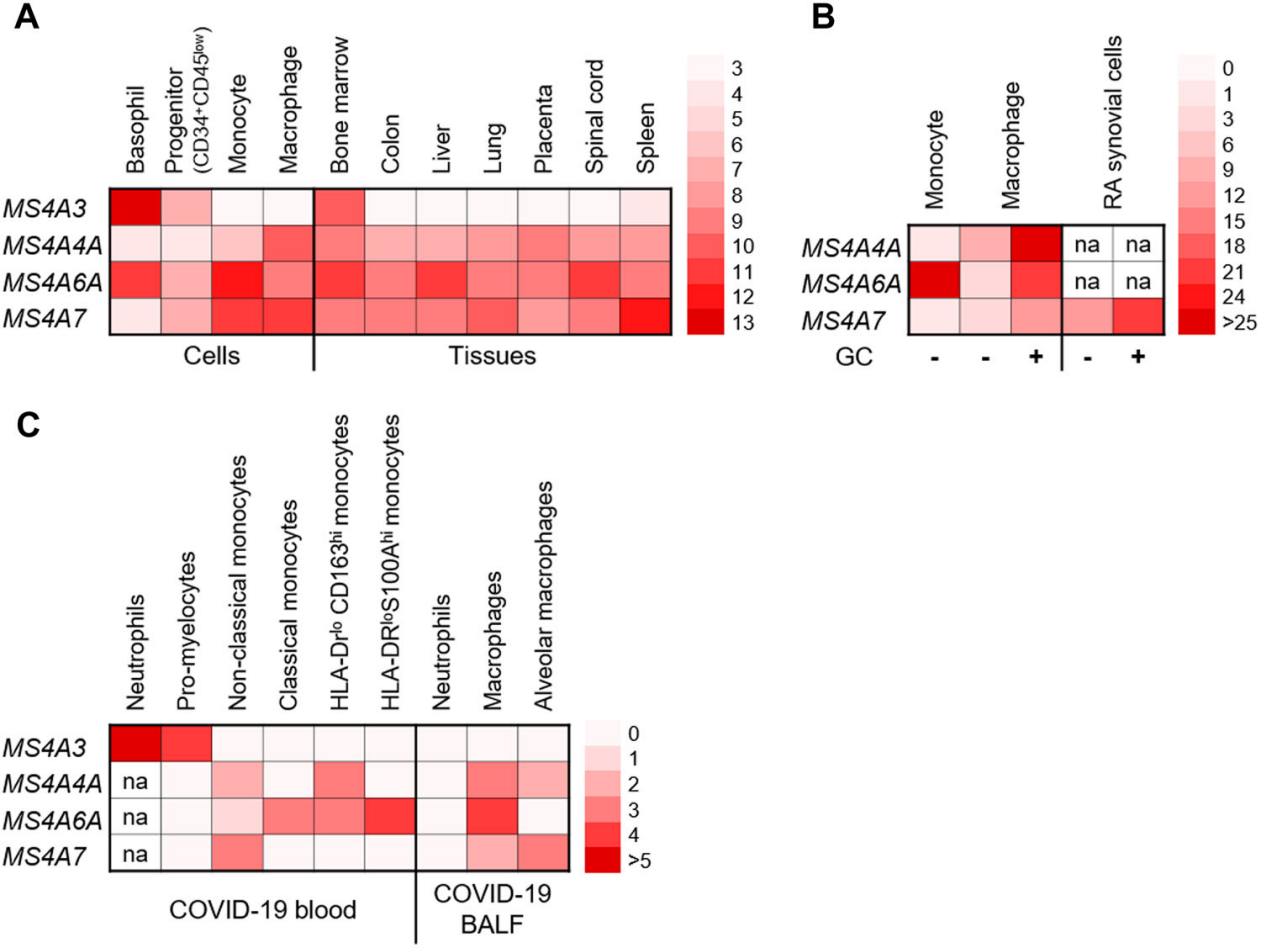
**Representative diagram of the main expression data of MS4A3, MS4A4A, MS4A6A, and MS4A7 in health and disease. (A)** Summary of RNA‐seq expression data in healthy cells and tissues. **(B)** Summary of PCR expression data in monocytes and Mϕs from healthy volunteers, glucocorticoid regulation in vitro and MS4A7 corticosteroid‐dependent upregulation in RA synovial tissue. **(C)** Summary of scRNA‐seq expression data in blood samples and BALF of COVID‐19 patients. **(A‐C)** Data were normalized by panel. na – not analyzed

We found that 7 members of the family (*MS4A2, MS4A4E, MS4A3, MS4A4A, MS4A6A, MS4A7*, and *MS4A14*) are prominently expressed in the myeloid lineage at different stages of differentiation. The finding that *MS4A3* and *MS4A4A* are expressed at the extremes of the myelomonocytic differentiation pathway (Fig. 12A and B), immature precursors and mature Mϕs, is consistent with previous findings.^9^, ^19^ In addition, we have observed that MS4A3 is part of the transcriptional signature of a cluster of immature “pro‐neutrophil” population that was reported to be increased in the blood of SARS‐CoV‐2 infected individuals with severe disease.^54^ By bulk‐RNA‐seq on purified circulating neutrophils from COVID‐19 patients, we obtained a similar finding. Interestingly, *MS4A3* is not detected in the neutrophil population found in the BALF of COVID‐19 patients. These observations suggest that MS4A3 is a candidate marker of circulating immature neutrophils enriched in COVID‐19 patients as a result of emergency myelopoiesis (Fig. 12C).^54^, ^64^ Surprisingly, contrarily to its low expression in healthy monocytes, *MS4A4A* was increased in specific “dysfunctional” monocyte subpopulations of COVID‐19 disease, calling for a further analysis to unveil its possible function in this disease.^54^, ^64^ Here, we also report that *MS4A6A* is highly expressed in CD14^+^ monocytes and is down‐regulated during M‐CSF driven monocyte‐to‐Mϕ differentiation. This pattern contrasts with that of *MS4A4A*, which is induced during Mϕ maturation. The analysis of RNA‐seq data from a public dataset confirmed the broad expression of *MS4A4A*, *MS4A6A*, and *MS4A7* in Mϕ‐enriched organs. Interestingly, these 3 genes were highly expressed in the spinal cord in comparison with the brain. This could be potentially related to a higher frequency of glial cells in the spinal cord compared to the brain,^65^ further confirming the restricted expression of these genes in Mϕ‐like cells.

The differential expression of MS4A tetraspan molecules in myelomonocytic cells suggests that they play an essential role in the specialized functions of these cells. Although very little is known about the function of MS4A proteins, it has been proposed that similarly to MS4A1 and MS4A2, other MS4A molecules could be involved in the intracellular Ca^2+^ concentration, and therefore cell activation.^10^, ^28^ Indeed, MS4A4A has been shown to regulate FcεRI signaling and Ca^2+^ entry in human mast cells.^26^ In the mouse, the Ms4a proteins expressed by necklace sensory olfactory neurons were reported to act as chemosensors for small ligands, regulating the influx of extracellular Ca^2+^.^66^ Additionally, MS4A12 is a colon‐restricted store‐operated calcium channel, which is involved in the malignant process.^67^, ^68^ Several tetraspanins, as CD9, CD63, CD81, or CD151, have been shown to interact with adhesion molecules, mediating the formation of endothelial adhesion platforms, and promoting leukocyte migration.^69^ Therefore, we speculate that members of MS4A family may play a role in cell differentiation and maturation.

Molecular partners have been identified for selected members of the MS4A family. MS4A2 is a component of the FcεRI and MS4A4A partners with Dectin‐1 and TREM‐2.^9^, ^16^, ^27^ Interestingly, MS4A4A co‐localizes with Dectin‐1 and TREM‐2 in the membrane of human Mϕs, and the expression of both MS4A4A and Dectin‐1 is co‐amplified by M2‐like signals.^9^, ^10^, ^23^, ^27^ In line with these data, we observed a positive correlation between the expression of *MS4A4A* and *CLEC7A* or *TREM2* genes in RNA‐seq analysis of human cells. Moreover, we have previously reported by FLIM‐FRET analysis that MS4A4A interacts with MS4A6A and MS4A7.^9^ Although we showed here that *MS4A6A* mRNA expression decreased during monocyte to Mϕ differentiation, the MS4A6A protein is still detectable on the membrane of mature Mϕs^9^ and therefore it is available for interacting with MS4A4A. Similar to tetraspanins, which operate in tetraspanin‐enriched domains,^70^ we hypothesized that the MS4A family proteins cluster in membrane associated platforms to regulate biological processes.^10^ However, the RNA stability and the translational regulation of MS4A proteins are largely unknown. The interactome of molecules of the MS4A family at different stages of myelomonocytic differentiation and activation remains to be fully defined.

Mϕs play a key role in neurodegeneration and strong genetic evidence links the *MS4A* locus to AD.^71^, ^72^, ^73^, ^74^, ^75^, ^76^ In particular, single nucleotide polymorphisms (SNPs) in the *MS4A6A* and *MS4A4A* genes have been shown to increase the risk of AD development.^71^, ^72^, ^73^, ^74^, ^75^, ^76^ In addition, SNPs in the *MS4A4A* gene were shown to regulate the production of soluble TREM‐2, an important microglia activator with a complex role in neurodegenerative disorders.^27^ Accordingly, we observed a similar expression pattern between *MS4A4A* and *TREM2* during monocyte‐to‐Mϕ differentiation in vitro. Thus, the expression fingerprint described here, the function of some members of the family and the disease genetic associations call for a systematic effort to unravel the function of MS4A tetraspan molecules.

*MS4A4A, MS4A6A, MS4A7*, and *MS4A4E* were found to be up‐regulated upon exposure to glucocorticoid hormones, confirming and extending previous data.^9^, ^58^, ^77^ Glucocorticoids diffuse through the plasma membrane and bind the glucocorticoid receptor (GR) in the cytosol. The GC‐GR complexes are transported into the nucleus where they regulate transcription by direct binding to glucocorticoid regulatory elements (GRE's) in the promoter region of glucocorticoid‐responsive genes or by binding other transcription factors.^78^ Indeed, Jubb et al. reported a dexamethasone dependent GR binding in the center of *Ms4a locus* in mouse Mϕs,^58^ and both *MS4A6A* and *MS4A4A* genes were shown to be up‐regulated by dexamethasone in human Mϕs.^9^, ^77^ The GR DNA binding sites in human monocyte‐derived Mϕs treated with dexamethasone overlapped with the binding‐site motifs of other transcription factors, namely PU.1, IRF4, and RXR,^77^ supporting the hypothesis of the involvement of these transcription factors in the regulation of *MS4A* genes. Indeed, by inspecting ChIP‐Seq tracks, only PU.1 peaks could be observed in concomitance with promoters of expressed genes.^48^, ^49^ A more accurate investigation of the transcriptional regulation of the MS4A genes might extend our knowledge on the function of MS4A proteins. *MS4A4A* expression was also increased after acute exposure to methylprednisolone, and was particularly high in Mϕs localized in the synovial tissue of RA patients.^9^ Similarly, an augmented expression of MS4A7 was observed in the synovial tissue of RA patients treated with glucocorticoids, further validating that MS4A proteins on Mϕs are regulated by glucocorticoids in vivo. The up‐regulation of several *MS4A* genes by anti‐inflammatory stimuli suggests an implication of MS4A proteins in the regulation of inflammation.^10^ mAbs targeting MS4A1/CD20 are invaluable tools in the treatment of B cells malignancies and autoimmunity. The role of Mϕ‐expressed MS4A family members in the activity of glucocorticoid hormones and their value as therapeutic targets deserves further studies.

The present study is a comprehensive analysis of the expression and regulation of members of the MS4A family in myeloid cells. The glucocorticoid‐dependent up‐regulation of MS4A members in Mϕs, their regulation during COVID‐19 and their implication in leukocyte differentiation and function, suggest a possible role in pathology. Thus, the value of MS4A proteins as targets or modulators in therapeutic approaches deserves further study.

## AUTHORSHIP

R.S.G. designed and conducted all the in vitro and *ex vivo* experiments, analyzed the data, prepared the figures, and wrote the manuscript. S.N.M. performed all the bioinformatics analysis and figure preparation, and edited the manuscript. M.A.B. performed the rheumatoid arthritis experiments, analyzed the data, and edited the manuscript. I.M. contributed to the experimental design, to the preparation of the manuscript and supported the scientific discussion. M.S. provided technical assistance in the in vitro experiments. F.G. prepared the tissue samples, supervised the IHC experiments, and revised the manuscript. F.C. performed the cell sorting. D.S. and S.C. conducted the neutrophil isolation and preparation for RNA‐seq. F.P. prepared the tissue samples and supervised the IHC experiments. M.S. and R.P. supported the scientific discussion and revised the manuscript. A.G. conducted the IHC in COVID‐19 tissue. C.P., M.L., and M.J.O. supported the scientific discussion and revised the manuscript. B.B. and A.M. supervised the study, wrote, and edited the manuscript.

Graduate Program in Areas of Basic and Applied Biology, Instituto de Ciências Biomédicas Abel Salazar, Universidade do Porto, Porto, Portugal.

Rita Silva‐Gomes and Sarah N. Mapelli, Barbara Bottazzi, Alberto Mantovani contributed equally to this work.

## DISCLOSURE

The authors declare no conflict of interest.

## Supporting information



Supplementary material

Supplementary material
