# Supplementary material for: Differential expression and regulation of MS4A family members in myeloid cells in physiological and pathological conditions
Source: J Leukoc Biol. 2021 Aug 4;111(4):817–36. doi: 10.1002/JLB.2A0421-200R (PMC9290968; doi:10.1002/JLB.2A0421-200R)

Supplementary Fig 1

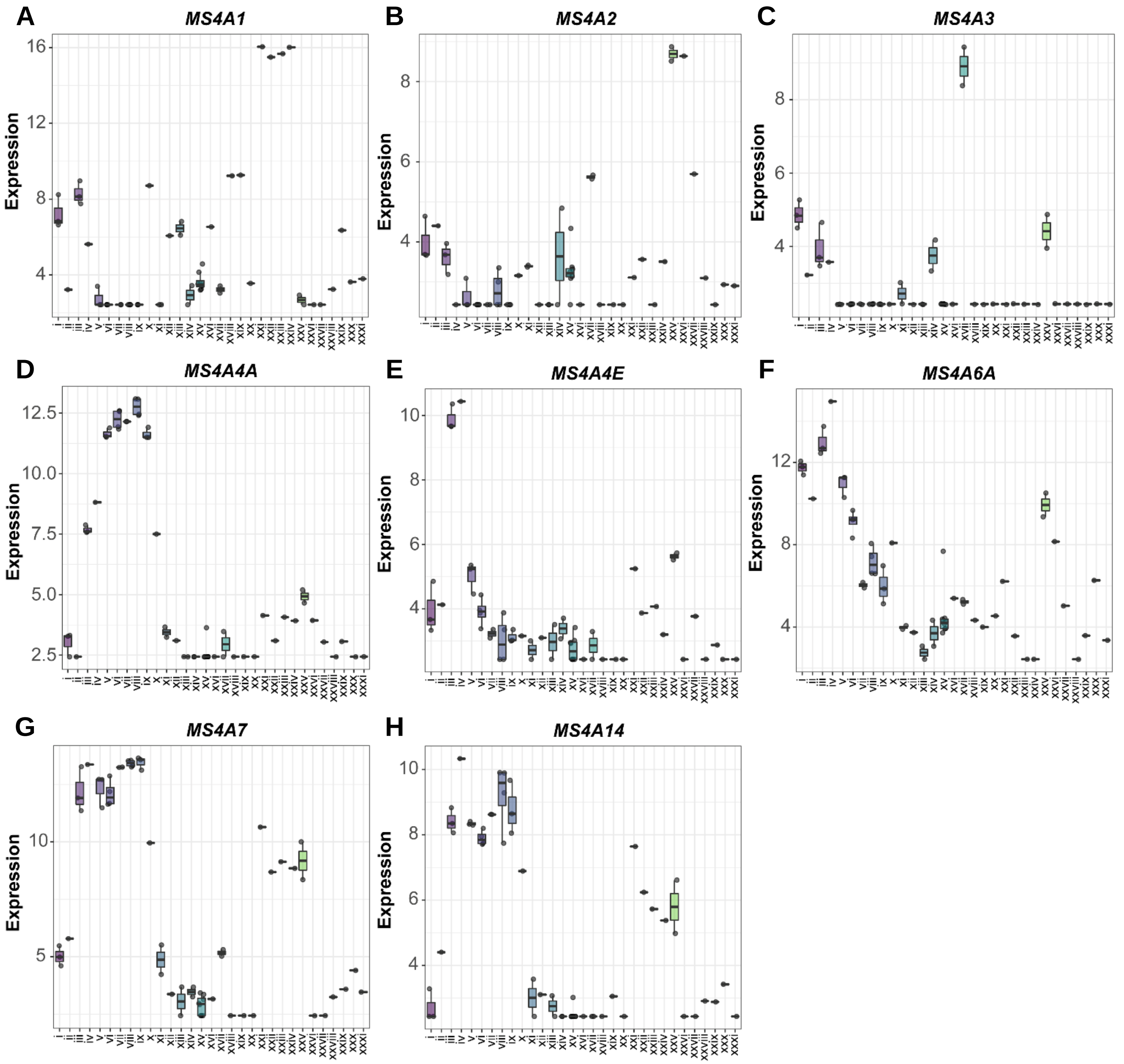

i – Cord Blood Mature Neutrophil
ii – Venous Blood Mature Neutrophil
iii – Cord Blood CD14$^+$ CD16$^-$ Monocyte
iv – Venous Blood CD14$^+$ CD16$^-$ Monocyte
v – Cord Blood Macrophage
vi – Venous Blood Macrophage
vii – Cord Blood Alternatively Activated Macrophage
viii – Venous Blood Alternatively Activated Macrophage
ix – Venous Blood Inflammatory Macrophage
x – Cord Blood Conventional DC
xi – Cord Blood CD34$^-$ CD41$^+$ CD42$^+$ Megakaryocyte
xii – Cord Blood Cytotoxic CD56^{dim} Nk Cell
xiii – Venous Blood Cytotoxic CD56^{dim} Nk Cell
xiv – Cord Blood CD4$^+$ αβ T Cell
xv – Venous Blood CD4$^+$ αβ T Cell
xvi – Venous Blood Effector Memory CD4$^+$ αβ T Cell
xvii – Cord Blood CD8$^+$ αβ T Cell
xviii – Venous Blood Central Memory CD8$^+$ αβ T Cell
xix – Venous Blood Effector Memory CD8$^+$ αβ T Cell
xx – Venous Blood Regulatory T Cell
xxi – Cord Blood CD38$^-$ Naïve B Cell
xxii – Venous Blood CD38$^-$ Naïve B Cell
xxiii – Venous Blood Memory B Cell
xxiv – Venous Blood Class Switched Memory B Cell
xxv – Cord Blood Erythroblast
xxvi – Umbilical Vein Resting Endothelial Cell
xxvii – Umbilical Vein Proliferating Endothelial Cell
xxviii – CD3$^-$ CD4$^+$ CD8$^+$ Thymocyte
xxix – CD3$^+$ CD4$^+$ CD8$^+$ Thymocyte
xxx – CD4$^+$ αβ Thymocyte
xxxi – CD8$^+$ αβ Thymocyte

Fig S 1 – Expression of MS4A family genes in human leukocytes and other cells. A – *MS4A1*, **B –** *MS4A2*, **C –** *MS4A3*, **D –** *MS4A4A*, **E –** *MS4A4E*, **F –** *MS4A6A*, **G –** *MS4A7*, **H –** *MS4A14*. RNAseq data was retrieved from *Chen L. et al. Cell (2016)* (EGAS00001000284 and EGAS00001000327). Expression is represented as Log2 of the normalized expression value.

Supplementary Fig 2

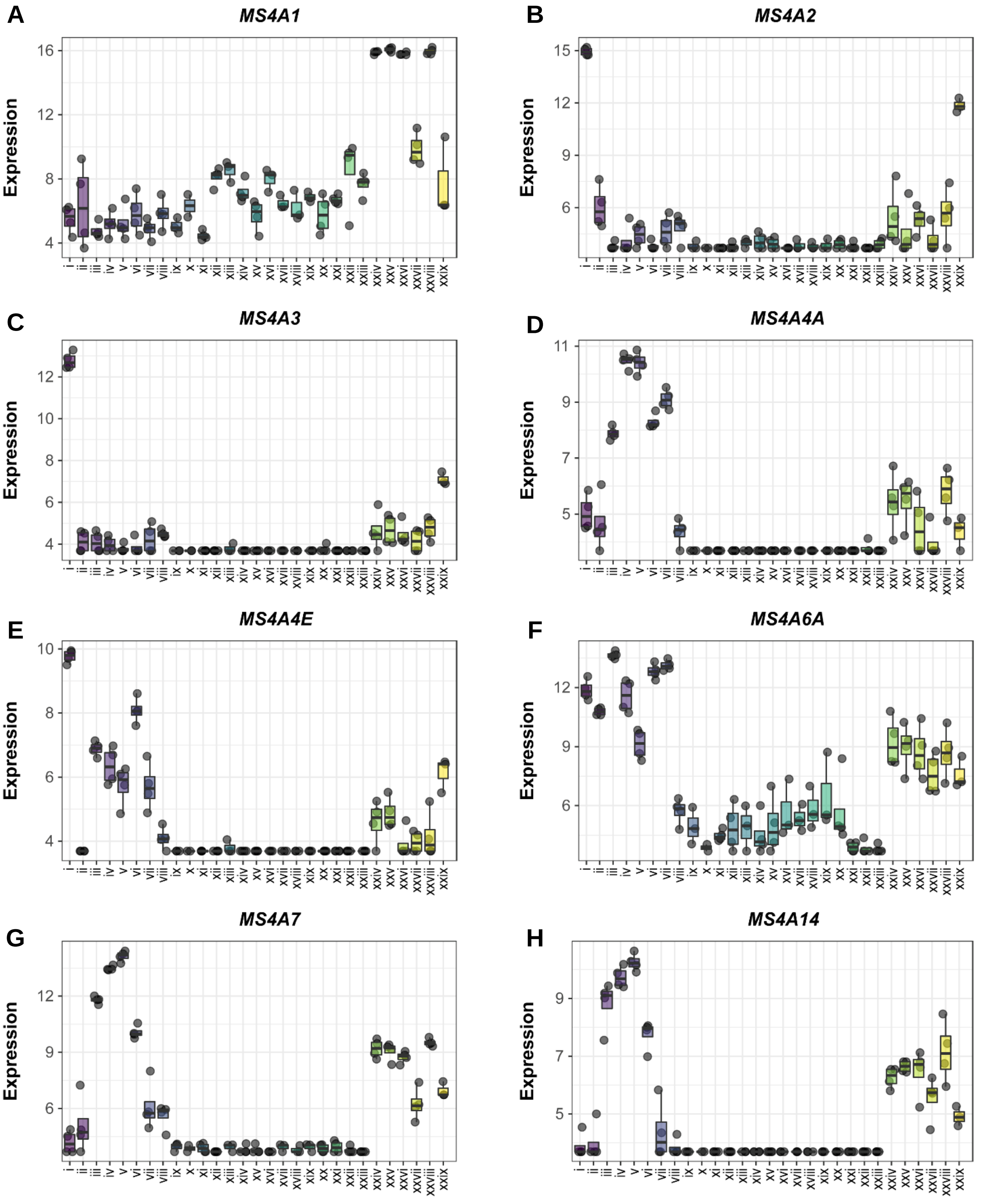

i – Low-density Basophils
ii – Low-density Neutrophils
iii – Classical Monocyte
iv – Intermediate Monocyte
v – Non-classical Monocytes
vi – Myeloid DCs
vii – Plasmatocytoid DCs
viii – NK Cells
ix – Naïve CD4 T Cells
x – Terminal effector CD4 T Cells
xi – Naïve CD8 T Cells
xii – Central Memory CD8 T Cells
xiii – Effector memory CD8 T Cells
xiv – Terminal Effector CD8 T Cells
xv – Follicular T Helper Cells
xvi – Th1 Cells
xvii – Th1-Th17 Cells
xviii – Th17 Cells
xix – Th2 Cells
xx – T Regulatory Cells
xxi – Non-Vd2 γδ T Cells
xxii – Vd2 γδ T Cells
xxiii – MAIT Cells
xxiv – Naïve B Cells
xxv – Non-switched memory B Cells
xxvi – Switched memory B Cells
xxvii – Plasmablasts
xxviii – Exhausted B Cells
xxix – Progenitor Cells

Fig S 2 – Expression of MS4A family genes in human leukocytes. A – *MS4A1*, **B** – *MS4A2*, **C** – *MS4A3*, **D** – *MS4A4A*, **E** – *MS4A4E*, **F** – *MS4A6A*, **G** – *MS4A7*, **H** – *MS4A14*. RNAseq data was retrieved from *Monaco G. et al. Cell Reports (2019)* (GSE107011). Expression is represented as Log2 of the normalized expression value.

Supplementary Fig 3

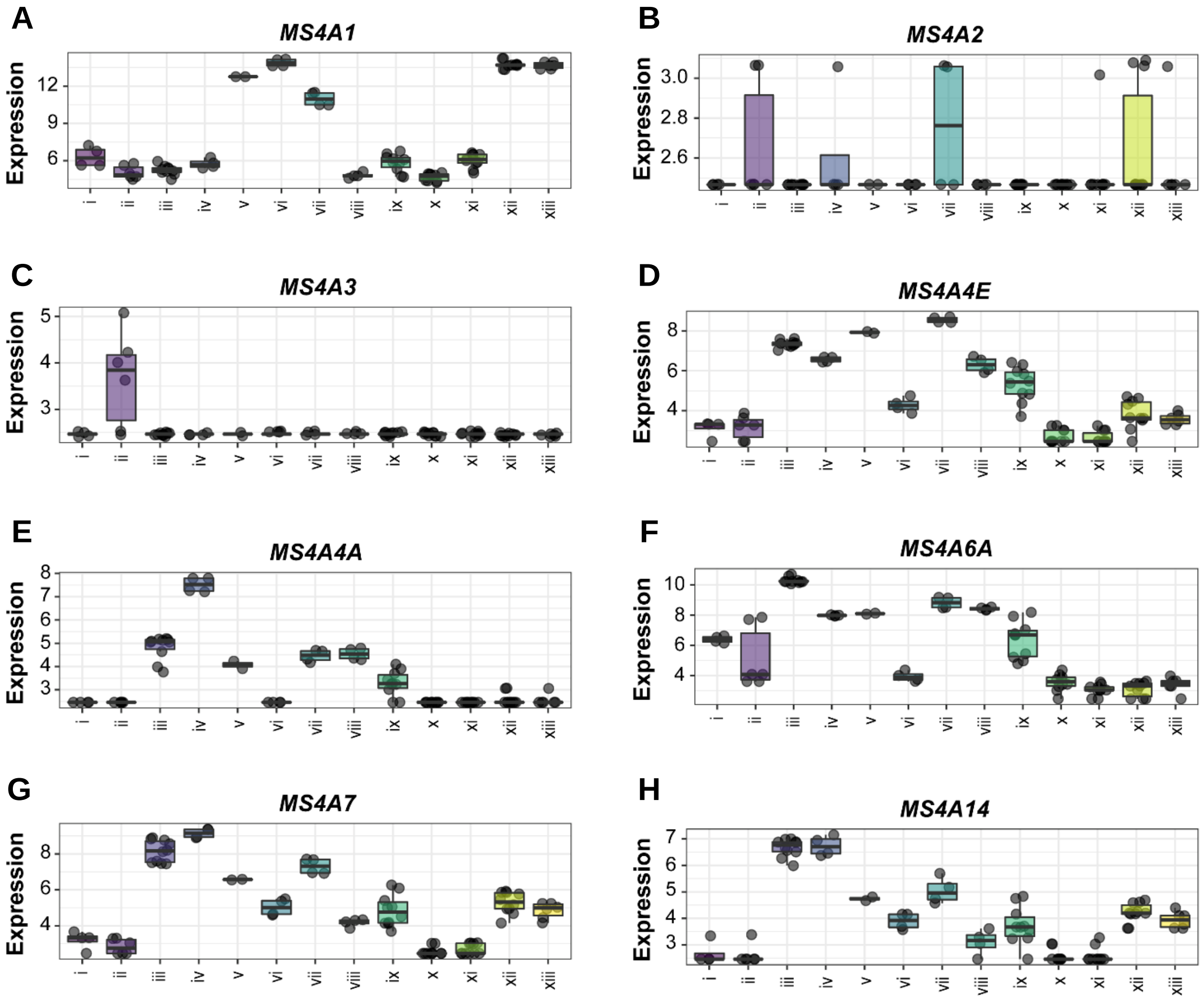

i – Eosinophils
ii – Neutrophils
iii – CD14^{Hi} BDCA1^{-} BDCA2^{-} CD123^{Lo}
iv – CD14^{Int} BDCA1^{-} BDCA2^{-} CD123^{Int}
v – CD14^{-} BDCA1^{+} BDCA2^{-} CD123^{-}
vi – CD3^{-} CD14^{-} BDCA2^{-} BDCA1^{+} CD123^{-}
vii – CD3^{-} CD14^{-} BDCA2^{-} BDCA1^{+} CD123^{+}
viii – CD14^{-} BDCA1^{-} BDCA2^{+} CD123^{Hi}
ix – CD3^{-} CD56^{+}
x – CD3^{+} CD19^{-} CD56^{-} CD4^{+}
xi – CD3^{+} CD19^{-} CD56^{-} CD8^{+}
xii – CD3^{-} CD19^{+} CD27^{Lo}
xiii – CD3^{-} CD19^{+} CD27^{Int}

Fig S 3 – Expression of MS4A family genes in human leukocytes. A – *MS4A1*, **B** – *MS4A2*, **C** – *MS4A3*, **D** – *MS4A4A*, **E** – *MS4A4E*, **F** – *MS4A6A*, **G** – *MS4A7*, **H** – *MS4A14*. RNAseq data was retrieved from *Choi J. et al. Nucleic Acids Res (2019)* (GSE115736). Expression is represented as Log2 of the normalized expression value.

Supplementary Fig 4

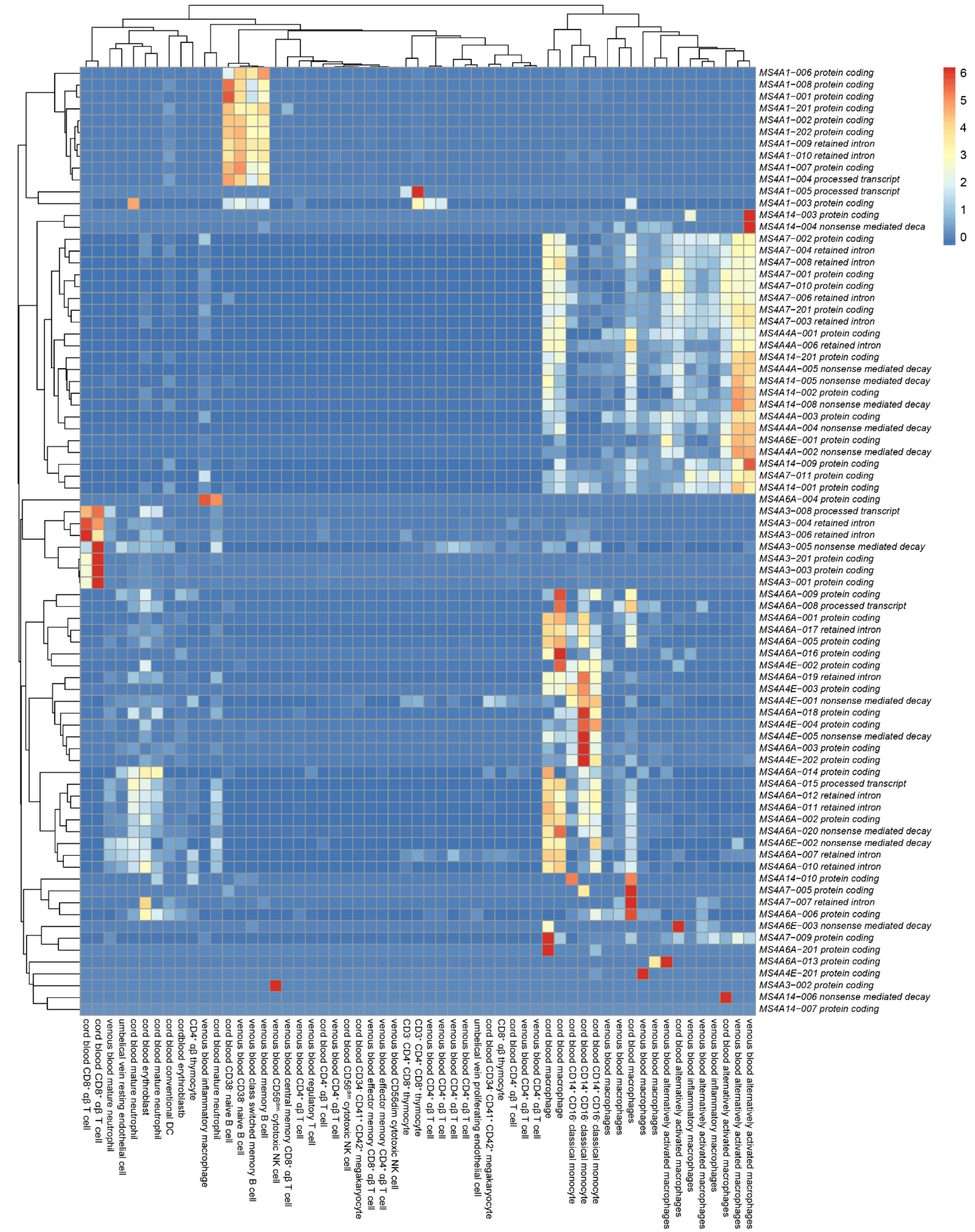

Fig S 4 – Expression of *MS4A1*, *MS4A3*, *MS4A4A*, *MS4A4E*, *MS4A6A*, *MS4A7* and *MS4A14* gene isoforms in common and rare human hematopoietic cells. RNAseq data was retrieved from *Chen L. et al. Cell (2016)* (EGAS00001000284 and EGAS00001000327). Data is represented as normalized TPM, and blue to red colors represent low to high expression, respectively. Double clustering analysis was performed by cell type and isoform.

Supplementary Fig 5

A

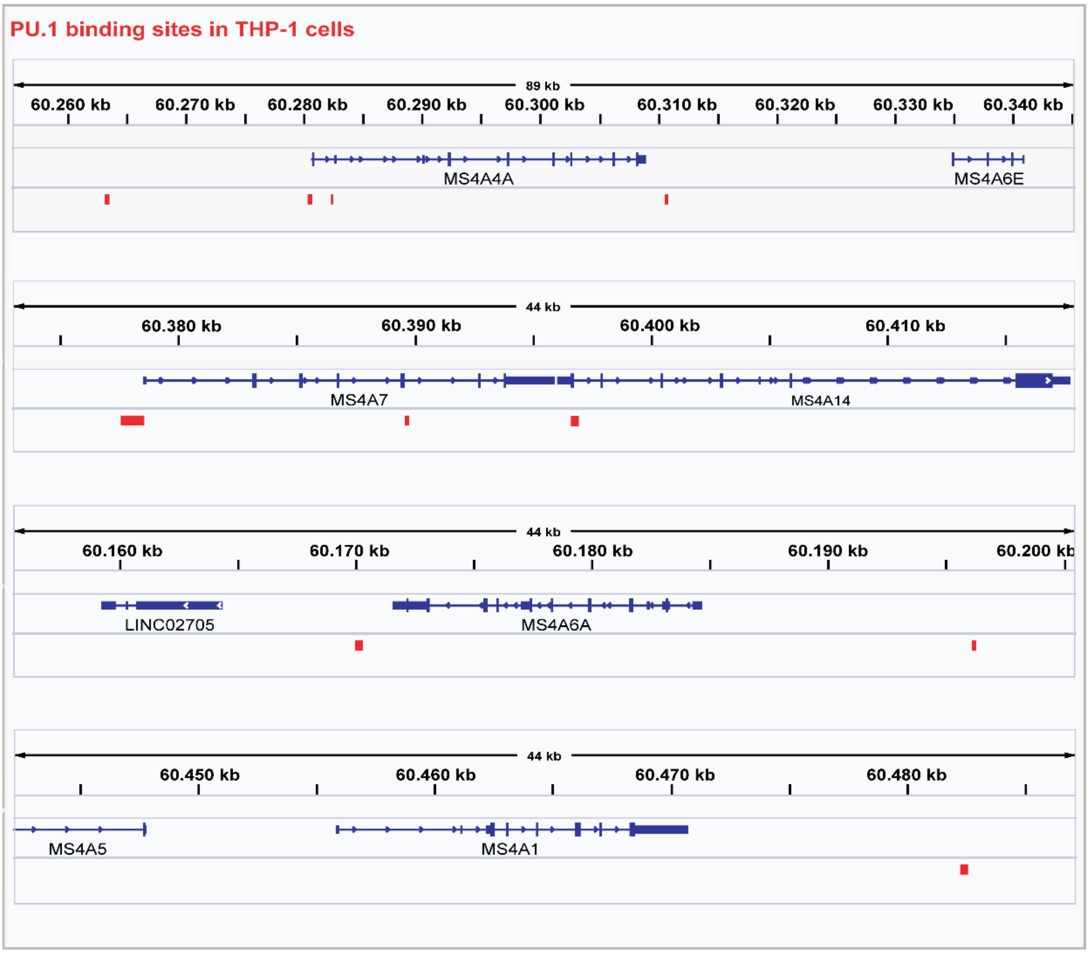

B

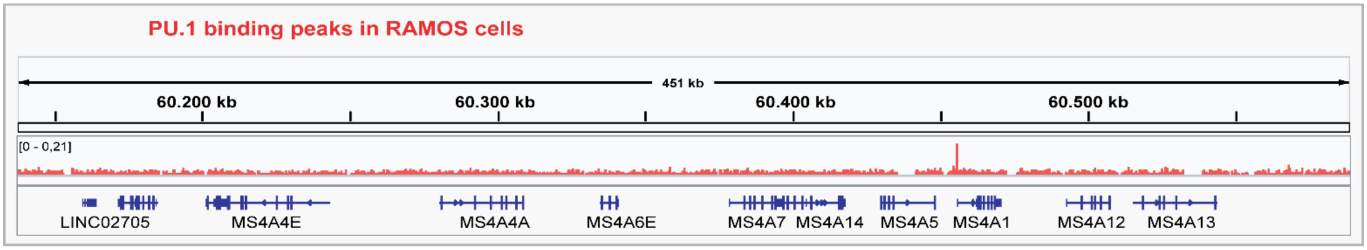

Fig S 5 – PU.1 binding sites and peaks derived from ChIP-Seq. (A) Predicted PU.1 binding sites in THP-1 cell line are reported in red and show selective enrichment in concomitance with proximal promoters of actively transcribed genes (*MS4A4A*, *MS4A7* and *MS4A14*). **(B)** PU.1 ChIP-Seq peaks in RAMOS cell line are reported as bigwig track. Along the genomic locus hosting *MS4A* family, a unique peak in *MS4A1* can be observed. ChIP-Seq data were retrieved from the work of **(A)** *Pott et al. PLoS One (2012)*, under the accession ID GSE25426, and **(B)** *Senigl et al. Cell Rep (2019)*, accession ID GSE139810.

Supplementary Fig 6

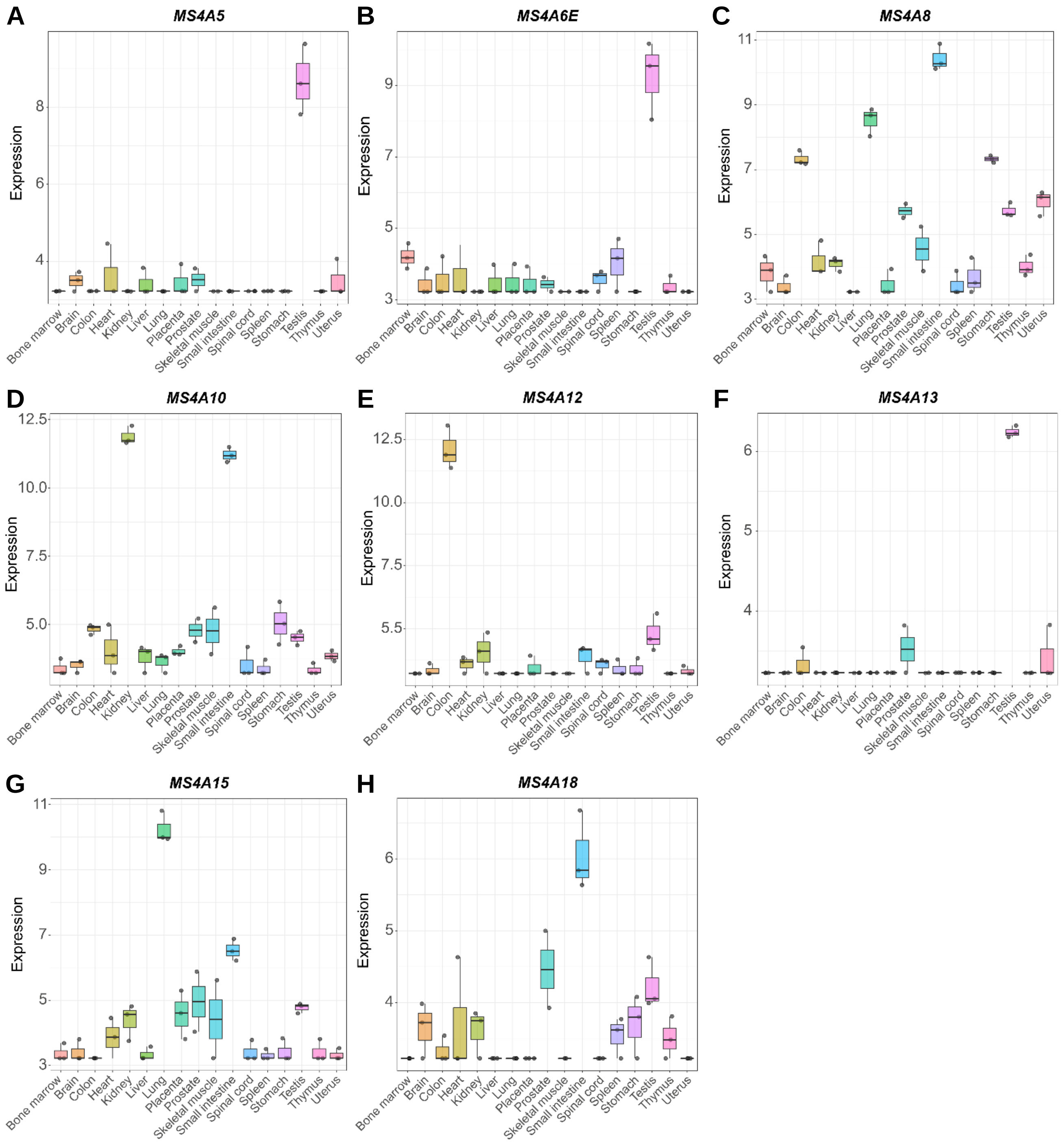

Fig S 6 - Expression of *MS4A* genes in human healthy tissues. Expression of *MS4A5* **(A)**, *MS4A6E* **(B)**, *MS4A8* **(C)**, *MS4A10* **(D)**, *MS4A12* **(E)**, *MS4A13* **(F)**, *MS4A15* **(G)** and *MS4A18* **(H)** in different human healthy tissues. RNA expression data was retrieved from *Ji P. et al. Cell Reports (2019)* (https://bigd.big.ac.cn/gsa/browse/CRA000348). Expression is represented as Log2 of the normalized expression value. Each dot represents one of three different RNA isolation methods for the same sample, including total RNA, poly(A) enrichment and RNase R treated RNA.

Supplementary Fig 7 (Graphical Abstract)

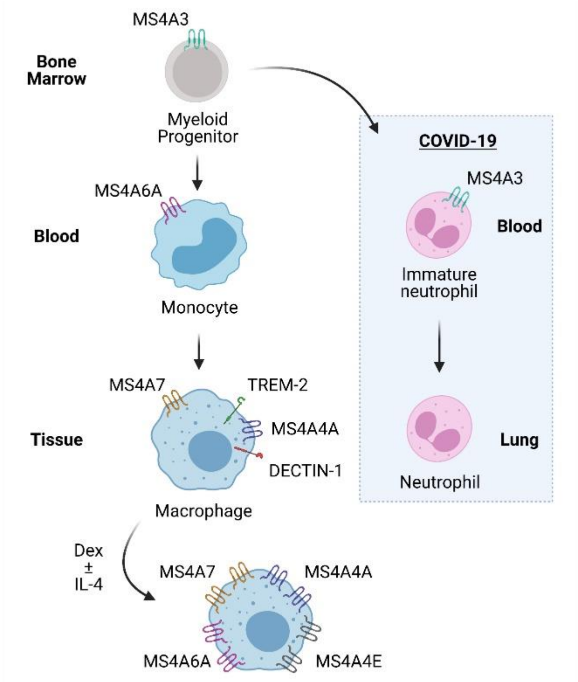

Supplement: Supplementary file 1 — Supplementary material [file JLB-111-817-s002.pdf]
