# Supplementary material for: Differential expression and regulation of MS4A family members in myeloid cells in physiological and pathological conditions
Source: J Leukoc Biol. 2021 Aug 4;111(4):817–36. doi: 10.1002/JLB.2A0421-200R (PMC9290968; doi:10.1002/JLB.2A0421-200R)
Supplement: Supplementary file 2 — Supplementary material [file JLB-111-817-s001.pdf]

Supplementary Note

Surname	Name
Anfray	Clement
Belgiovine	Cristina
Bertocchi	Alice
Bombace	Sara
Brescia	Paola
Calcaterra	Francesca
Calvi	Michela
Cancellara	Assunta
Capucetti	Arianna
Carenza	Claudia
Carloni	Sara
Carnevale	Silvia
Cazzetta	Valentina
Coianiz	Nicolò
Darwich	Abbass
De paoli	Federica
Di donato	Rachele
Digifico	Elisabeth
Durante	Barbara
Farina	Floriana
Ferrari	Valentina
Fornasa	Giulia
Franzese	Sara
Gil Gomez	Antonio
Giugliano	Silvia
Gomes	Ana Rita
Lizier	Michela
Lo Cascio	Antonino
Melacarne	Alessia
Mozzarelli	Alessandro
My	Ilaria
Oresta	Bianca
Pasqualini	Fabio
Pastò	Anna
Pelamatti	Erica
Perucchini	Chiara
Pozzi	Chiara
Rimoldi	Valeria
Rimoldi	Monica
Scarpa	Alice
Silvestri	Alessandra
Sironi	Marina
Spadoni	Ilaria
Spano	Salvatore
Spata	Gianmarco
Supino	Domenico
Tentorio	Paolo
Ummarino	Aldo
Valentino	Sonia
Zaghi	Elisa
Zanon	Veronica
